# One-Step Electropolymerization of a Dicyanobenzene-Carbazole-Imidazole Dye to Prepare Photoactive Redox Polymer Films

**DOI:** 10.3390/polym15163340

**Published:** 2023-08-08

**Authors:** Jinkun Liu, Octavio Martinez Perez, Dominic Lavergne, Loorthuraja Rasu, Elizabeth Murphy, Andy Galvez-Rodriguez, Steven H. Bergens

**Affiliations:** Department of Chemistry, University of Alberta, 11227 Saskatchewan Drive, Edmonton, AB T6G 2G2, Canada

**Keywords:** 4CzIPN, 3CzImIPN, photoredox-POP, photochemistry, polymerization

## Abstract

To the best of our knowledge, this study reports the first direct electropolymerization of a dicyanobenzene-carbazole dye functionalized with an imidazole group to prepare redox- and photoactive porous organic polymer (POP) films in controlled amounts. The POP films were grown on indium-doped tin oxide (ITO) and carbon surfaces using a new monomer, 1-imidazole-2,4,6-tri(carbazol-9-yl)-3,5-dicyanobenzene (**1**, 3CzImIPN), through a simple one-step process. The structure and activities of the POP films were investigated as photoelectrodes for electrooxidations, as heterogeneous photocatalysts for photosynthetic olefin isomerizations, and for solid-state photoluminescence behavior tunable by lithium-ion concentrations in solution. The results demonstrate that the photoredox-POPs can be used as efficient photocatalysts, and they have potential applications in sensing.

## 1. Introduction

Molecular dyes are utilized in applications that include photocatalysis of organic reactions, as components of OLED (organic light-emitting devices) displays, in photodynamic cancer therapy, in dye-sensitized solar cells, and in photoelectrodes for solar fuels [1,2]. Ru- [3,4,5] and Ir-polypyridyl [6,7] and related complexes are common molecular dyes because they have strong absorption in the visible range (metal-to-ligand charge transfer). Moreover, they are readily modified to tune the wavelengths of absorption and emission as well as the redox properties of the excited state, and they readily undergo intersystem crossing to form relatively long-lived triplet excited states. There are inherent disadvantages with these compounds that include cost, toxicity, and low stability under certain reaction conditions that limit their large-scale application [8,9,10]. Organic dyes that are abundant and easy to prepare offer a promising alternative to precious-metal-based systems [11,12,13]. The most studied organic dyes include perylene derivatives [14], porphyrins [15], triphenylamines [16], and subporphyrins [17]. The excitation of push–pull dyes involves intramolecular charge transfer [3,4,5]. The push–pull dye 1,2,3,5-tetrakis(carbazol-9-yl)-4,6-dicyanobenzene (4CzIPN) [18,19,20] has been successfully utilized in OLEDs [21,22,23] and as photocatalysts for organic reactions [24,25,26,27,28], the hydrogen photoevolution reaction [29], and CO_2_ photoreduction [30,31].

Our group and others have attached organic dyes to electrode surfaces by electrografting with diazonium precursors [32,33,34]. The resulting carbon (dye)–oxygen (semiconductor) bond shows promising stability under alkaline conditions that exceed those of the carboxylate or phosphonate bridges typically used to attach molecular chromophores to semiconductors [32,35]. We previously reported the electrografting of 4CzIPN onto indium-doped tin oxide (ITO) or carbon electrodes using diazonium chemistry, and the system was more stable and active under basic conditions than the representative Ru-polypyridyl complexes [36].

Porous organic polymers (POPs) offer several potential advantages over monolayers or near-monolayers of organic dyes attached to electrode surfaces [37]. For example, POPs have three-dimensional, large surface-area (i.e., high specific surface-area) structures that may coordinate with redox catalysts or act as sensor detection sites. In addition, 3-dimensional POPs made of dye monomers offer larger cross sections for light absorption than their corresponding monolayers. As well, many POPs are grown directly from simple organic monomers that do not require synthetic modifications, greatly simplifying the preparation, processing, and purification steps for large-scale preparations [28,38]. Carbazole-containing molecules have been extensively studied as electropolymerization monomers, and a wide variety of polycarbazole electropolymers have been developed for purposes that include OLEDs, capacitors, and memory devices [39]. To our knowledge, there is one example of a POP made from a dicyanobenzene-carbazole chromophore [28]. In that report, the parent dye, 4CzIPN, was copolymerized with formaldehyde dimethyl acetal using an Fe(III)-redox oxidant to affect the polymerization. The resulting polymer was evaluated as a heterogeneous photocatalyst for construction of C(sp^3^)–P bonds and selective oxidation of sulfides in water under mild conditions [28].

We now report the first direct electropolymerization of a dicyanobenzene-carbazole dye. Specifically, we prepared redox- and photoactive-POP films in controlled amounts employing a new monomer, namely, 1-imidazole-2,4,6-tri(carbazol-9-yl)-3,5-dicyanobenzene (**1**, 3CzImIPN), on flat ITO (**1**-ITO), on ITO nanoparticles (**1**-ITO-NP) and on carbon (**1**-CP) surfaces. The reasons we focused on 3CzImIPN includes not only its similarity to 4CzIPN in acting as a thermally activated delayed fluorescence (TADF) photo-active catalyst, but also due to the N-site linkage facilitated by the imidazole group. This linkage allows for potential interactions with other active metal molecular catalysts or metal ions, rendering it suitable for applications in photo-assisted CO_2_ reduction, N_2_ reduction, other photo-redox organic reactions, or ion sensing. We report the structure and activities of the POP films as photoelectrodes for electrooxidations, as heterogeneous photocatalysts for photosynthetic olefin isomerizations, and as solid-state fluorescent metal ion sensors.

## 2. Materials and Methods

Materials: Chemicals were used without any further treatment unless mentioned otherwise. The following compounds were purchased from Sigma Aldrich (Oakville, ON, Canada): hydroxypropyl cellulose (powder, 20 mesh particle size, MW ~100,000); tetrabutylammonium hexafluorophosphate (TBAPF_6_; for electrochemical analysis, ≥99.0%); hydroquinone (ReagentPlus, ≥99.5%); triethylamine, distilled (≥99.0%); acetonitrile, distilled (for HPLC, gradient grade, ≥99.9%); dichloromethane, distilled (DCM; ACS reagent, ≥99.5%); carbazole (≥95%); NaH (60% dispersion in mineral oil); NaCl and NaClO_4_ (ACS reagent, ≥98.0%); calcium hydride (reagent grade, 95%); and trans-stilbene (96%). ITO nanoparticles were purchased from Fisher Scientific (17–28 nm APS powder). Anhydrous ethanol was purchased from Greenfield Global (Chatham, ON, Canada). Tetrafluoroisophthalonitrile (>98.0%) was purchased from TCI chemicals (Portland, OR, USA). The solvents tetrahydrofuran (Na/benzophenone), toluene (CaH_2_), DCM (CaH_2_), and acetonitrile (CaH_2_) were dried by distillation from the appropriate drying agent under N_2_. Triple-distilled water was used for all glassware cleaning and preparation of aqueous solutions for electrochemistry experiments.

### 2.1. Fabrication of ITO Nanoparticle-Coated ITO Glass Electrode (ITO-NP)

Indium tin oxide-coated glass slides (ITO glass, Kaivo, surface resistivity <7 Ω/sq) were cut into 1 cm × 2.5 cm pieces and sonicated in ethanol, triple-distilled water, and acetone for 30 min each, followed by drying in an oven at 60 °C. ITO nanoparticle paste was prepared according to the following procedure: 1.32 g of hydroxypropyl cellulose (HPC) was placed in a vial with 15 mL of anhydrous ethanol and stirred overnight to obtain the HPC suspension. Next, 1.5 g of ITO nanoparticles was dispersed in 6.25 mL of anhydrous ethanol and sonicated for 40 min. After sonication, 3.75 mL of the HPC suspension was added to the ITO nanoparticle suspension and stirred overnight followed by sonicating for 1 h before use to prepare the ITO paste. The ITO paste was then doctor-bladed on an ITO surface with 4 layers of scotch tape as a spacer. After drying in air, the prepared electrodes were heated from room temperature to 500 °C over 1 h and then kept at 500 °C for another 1 h in a furnace. The ITO-NP electrodes were collected after the furnace cooled down to room temperature.

### 2.2. Preparation of Poly 3CzImIPN (**1**) on ITO-NP, ITO Glass (ITO), or Carbon Fiber Paper (CP)

The ITO glass was cut into 1 cm × 2.5 cm small pieces and then sonicated in triple-distilled water, ethanol, and acetone for 30 min, respectively. CP was also cut into 1 cm × 2.5 cm pieces and sonicated in triple-distilled water for 30 min. After drying in an oven at 60 °C for 30 min, the ITO or CF was ready to use for the next step. Electropolymerization was carried out in distilled dichloromethane (DCM) solution, with 1 mM of **1** and 0.1 M of TBAPF_6_. Electropolymerizations over ITO-NP electrodes were recorded by sweeping between 0 and 2 V vs. Ag wire (−0.55 to 1.45 V vs. Fc/Fc^+^, Fc = ferrocene) at a scan rate of 100 mV s^−1^ for 5, 10, or 45 cycles (5/10/45-**1**-ITO-NP), with Ag wire as the reference electrode and platinum gauze as the counter electrode. Electropolymerizations over ITO or CP electrodes were recorded by sweeping between 0 and 3 V vs. Ag wire (−0.55 to 2.45 V vs. Fc/Fc^+^) at a scan rate of 100 mV s^−1^ for 45 cycles (45-**1**-ITO or 45-**1**-CP), with Ag wire as the reference electrode and platinum gauze as the counter electrode. Ferrocene was added after the experiment as an internal reference. After polymerization, the electrodes were washed with DCM and dried in an oven at 60 °C for 45 min.

### 2.3. Stilbene Isomerization

In this experiment, *E*-stilbene (45.1 mg, 0.25 mmol) was introduced into a custom-made test tube with a magnetic stir bar and polymer **1** on an ITO-NP electrode. Subsequently, the test tube was inserted into a side arm flask, which was connected to a Schlenk line, evacuated, and re-filled with argon for three cycles. Freshly distilled toluene (2.5 mL) was then injected into the test tube using a gas-tight syringe under an argon atmosphere. Upon adding toluene, the resulting solution was stirred at room temperature under blue LED radiation with a fan. To monitor the reaction, 50 μL of solution drawn from the reaction vessel, and ^1^H NMR spectra were recorded using CDCl_3_ (deuterated chloroform) as a solvent. 1,3,5-Trimethoxy benzene was added as an internal standard at the end of the reaction.

### 2.4. Photoluminescence Response to Lithium Ions

The 45-**1**-CP electrode was first soaked in distilled water for 2 h. Subsequently, the electrode was taken out of the water without drying, and its photoluminescence spectrum was immediately measured (Figure 5c). Afterwards, the electrode was exposed to aqueous solutions containing 10^−4^ M and 10^−2^ M LiClO_4_, respectively, for 120 min, and the photoluminescence spectrum was measured immediately as described above (Figure 5c).

### 2.5. Characterization

Scanning electron microscopy (SEM) with energy-dispersive X-ray spectroscopy (EDX) mapping measurements were conducted using a Zeiss EVO MA10 scanning electron microscope with EDX (Jena, Germany). Typically, the system vacuum of SEM was lower than 2 × 10^−5^ torr when acquiring images. The X-ray photoelectron spectroscopy (XPS) measurements were conducted using a Kratos Axis Ultra (Kratos Analytical, Manchester, UK). A monochromatized Al Kα source (hν = 1486.71 eV) was used, while the pressure in the sample analytical chamber was maintained below 5 × 10^−10^ torr. Survey scans covered the binding energies of 1100–0 eV with 160 eV analyzer pass energy. For the deconvolution process, the spectra were calibrated to position C–C binding energy at 284.6 eV in order to correct the charge effect. Solid-state UV–Vis spectra were collected using a Cary 5000 UV–Vis (Santa Clarita, CA, USA) spectrometer in reflection mode. Solid-state photoluminescence was acquired using a Horiba-PTI QM-8075-11 (Edison, NJ, USA) fluorescence system with a solid stand. FTIR was measured using a Thermo Nicolet 8700 FTIR spectrometer and continuum FTIR microscope on ATR (Thermo Fischer Scientific Instruments, Waltham, MA, USA). The ^1^H NMR spectra were acquired using 400 MHz, 500 MHz, or 600 MHz Varian Inova or Varian DD2 M2 400 MHz NMR spectrometers (Agilent Technologies, Santa Clara, CA, USA). The ^13^C NMR spectra were acquired using a Varian VNMRS 500 MHz NMR spectrometer (Agilent Technologies, Santa Clara, CA, USA). The chemical shifts were reported in parts per million relative to TMS with the solvent as the internal standard. Abbreviations used in reporting of NMR data are s (singlet), d (doublet), t (triplet), q (quartet), dd (doublet of doublet), dq (doublet of quartet), and m (multiplet). HRMS spectra were acquired using either electrospray ionization in an Agilent 6220 ao TOF mass spectrometer (Agilent Technologies, Santa Clara, CA, USA) or electron ionization on a Kratos Analytical MS50G double-focusing sector mass spectrometer (Kratos Analytical, Ltd., Manchester, UK). The experiment involving simulated sunlight was carried out using a 300 W xenon light source solar simulator equipped with an AM 1.5G optical filter (Xian Toption Instrument Co., Ltd., Xi’an, Shaanxi, China). To obtain a cross-sectional view of the film using a SEM, the sample was first coated with gold as the conduction layer and then coated with tungsten as the protection layer. The measured angle was autocorrected to obtain the thickness of the polymer film.

## 3. Results and Discussion

### 3.1. Preparation of Monomer

**Scheme 1:**
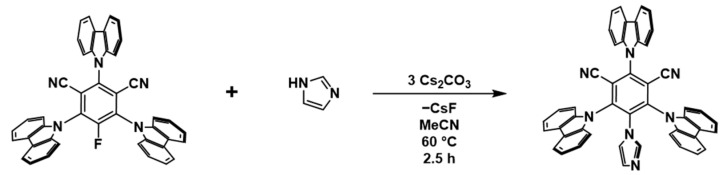
Synthesis of the monomer 3CzImIPN (**1**).

The imidazole-dye compound **1** was prepared by displacement of fluoride in the known precursor 1-fluoro-2,4,6-tri(carbazol-9-yl)-3,5-dicyanobenzene by cesium imidazolate in 95% yield (Scheme 1).

**Figure 1:**
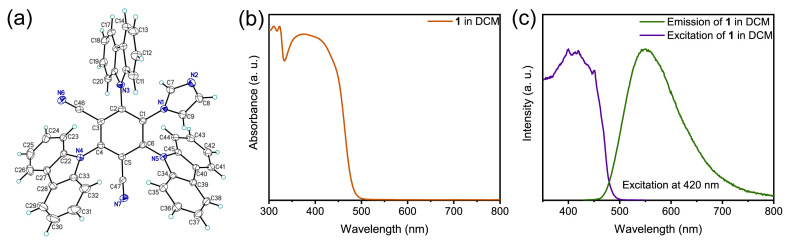
(**a**) The X-ray structure of **1**, (**b**) the absorbance UV–Vis spectrum of **1** (0.1 mM) in dichloromethane (DCM), and (**c**) the excitation and emission curves of **1** (of 0.1 mM) in DCM.

Figure 1a shows the solid-state structure of **1**. As has been observed in related compounds [40,41], the dihedral angles between the benzene and carbazole rings range from 60–70° to relieve steric crowding. The dihedral angle between the benzene and imidazole ring is ~52°. Figure 1b shows the UV–Vis and steady-state photoluminescence spectra recorded in dichloromethane. As is well known for carbazole-dicyanobenzene dyes [40], the excitation at ~421 nm results in a strong emission at ~543 nm. In keeping with the known photochemistry of this class of compound, the excitation is a charge-transfer excitation from the highest occupied molecular orbital (HOMO), largely located on the carbazole rings, to the lowest unoccupied molecular orbital (LUMO), largely centered on the dicyanobenzene moiety. The S_1_ and T_1_ excited states are typically close in energy for this class of compounds, contributing to rapid intersystem crossing. Further, the T_1_ excited state is sufficiently long-lived to promote photoredox and energy-transfer organic photoreactions [42,43].

### 3.2. Electropolymerization of 3CzImIPN (**1**)

**Figure 2:**
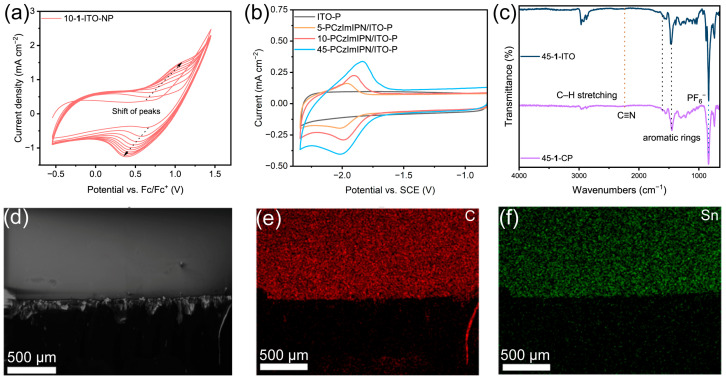
(**a**) CV curves of 10-**1**-ITO-NP in 0.1 M TBAPF_6_ and 1 mM of **1**. Sweep rate 100 mV s^−1^, 10 sweeps, sweep range from −0.55 to 1.45 vs. Fc/Fc^+^. The dashed arrows indicate direction of peak potential shift as the sweep # increases. (**b**) CV (sweep rate 10 mV s^−1^; 5, 10, 45 sweeps; sweep range from −0.41 to −1.91 vs. Fc/Fc^+^) curves of 5, 10, and 45-**1**-ITO-NP compared with ITO-P in 0.1 M TBAPF_6_ acetonitrile solution without monomer. (**c**) FT-IR of 45-**1**-ITO and 45-**1**-CP. (**d**) Tilted SEM of 45-**1**-ITO and corresponding (**e**) C and (**f**) Sn EDX mapping on ITO glass.

The electropolymerization of carbazole-containing molecules is well known [44,45,46]. Briefly, the carbazole ring undergoes one-electron oxidation to form the corresponding cationic radical, followed by carbon–carbon bond formation at the 3- or 6-positions. Proton loss then forms the neutral polymer. Figure 2a shows the cyclic voltammograms (CVs) for the 10-cycle potentiodynamic electropolymerization of **1** over ITO nanoparticles on ITO glass (the resulting polymer is designated as 10-**1**-ITO-NP) carried out in CH_2_Cl_2_ solution under air (0.1 M tetrabutyl ammonium hexafluorophosphate (TBAPF_6_), 1.0 mM **1**, sweep rate = 100 mV s^−1^, 10 sweeps, and sweep range = −0.55 to 1.45 vs. ferrocene/ferrocene^+^ (Fc/Fc^+^, unless stated otherwise, all potentials in this paper are relative to Fc/Fc^+^)). The first positive-going sweep contains a strong anodic oxidation that commences at ~0.75 V vs. Fc/Fc^+^ that corresponds to the one-electron oxidation of a carbazole ring (at nitrogen) to form the cationic radical that then undergoes electropolymerization.

**Scheme 2:**
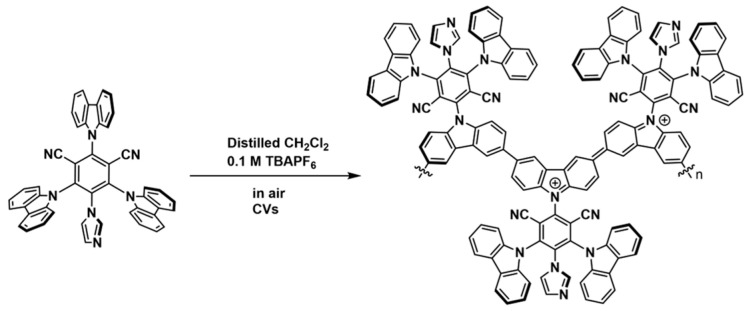
Proposed structure of the polymer in the neutral and oxidized (doped) forms. This redox behavior is typical of polycarbazoles [39,49,50].

The electropolymerization continues as the potential increases. The first negative-going sweep contains a reductive wave at ~0.6 V that corresponds to the electroreduction of the POP deposited on the electrode during the first sweep. The second positive-going sweep contains a new peak at ~0.7 V that corresponds to the electrooxidation of the POP. Scheme 2 shows the proposed structure of the POP in both the reduced (neutral) and oxidized (polycationic) forms. The oxidative wave at ~0.7 V specifically arises from 1 e^−^ oxidation of the carbazole-nitrogen centers in the polymer to form the polycationic, conjugated polymer shown in Scheme 2. This behavior is typical of polycarbazoles, and the conjugated polycationic polymers are known to be electronically conductive. The reductive wave at ~0.6 V corresponds to the reduction of the positive nitrogen centers in the polycation to form the neutral polycarbazole structure shown in Scheme 2. These CVs are similar to those in the previous report by Li and his colleagues when obtaining polymers of carbazole derivatives [47]. Furthermore, the use of high upper sweep limits, as was done during these experiments, is known to produce polycarbazoles, not simple dimers [47]. Again, this behaviour is quite typical of polycarbazoles. Scheme 2 illustrates the results from the polymerization occurring at one carbazole ring per molecule of **1**. In principle, the polymerization can occur at more than one carbazole ring. Further, Scheme 2 illustrates the polymerization occurring at both the 3- and 6-positions of the same carbazole ring. In principle, however, polymer growth can occur at more than one carbazole ring and at only one of the 3- or 6-positions. We note, however, that sweeping to high potentials typically results in polymerization at both positions [47]. The amount of the resulting polymer increases with each subsequent sweep, as shown by the increase in charge under the redox peaks for the polymer, and their potential shifts. Specifically, the cathodic peak shifts from ~0.65 V in the first negative-going sweep to more reducing potentials, and the anodic peak shifts to more oxidizing potentials as the amount of polymer increases, slowing the rate of electron and ion transfer through the POP. The resulting POP (10-**1**-ITO-NP) contains redox-active groups in the backbone and photoactive groups in the polymerized monomer, and it is capable of reactions at the imide groups. The degree of crosslinking in the photoredox-POP is unknown. The film transparency at 600 nm of 45-**1**-ITO was 70.4. The film thickness was 362 nm, proving it is not a simple dimer (Figure S11 in Supplementary Materials).

Similar trends were observed during the 5- and 45-cycle electropolymerizations forming the 5-**1**-ITO-NP and 45-**1**-ITO-NP, respectively (Figure S1). Figure 2b shows the CV curves sweeping to negative potentials (sweep rate 10 mV s^−1^; 5, 10, 45 sweeps; sweep range from -0.41 to -1.91 vs. Fc/Fc^+^) for the isolated 5-, 10-, and 45-**1**-ITO-NP photoredox-POPs recorded in acetonitrile solution under N_2_ (0.1 M TBAPF_6_). The electrodes were removed from the electropolymerization solution and washed thoroughly with CH_2_Cl_2_ before the CVs were recorded. The photoredox-POPs all contain highly reversible reduction peaks at ~−1.6 V vs. Fc/Fc^+^ [48]. The corresponding oxidation peak occurs ~−1.5 V in the positive-going sweep. The CV of the free monomer **1** contains the corresponding 1 e^−^ reversible redox wave at ~−1.5 V, and it corresponds to the 1 e^−^ reduction and oxidation of the dicyanobenzene ring [36]. The CVs of the electrodes (Figure 2b and Figure S2 in Supplementary Materials) show that the dicyanobenzene rings in the photoredox-POPs undergo the same redox process. The charge under these peaks increased with increasing electropolymerization cycles, showing that the amount of the photoredox-POP is controlled by the number of potential sweeps during the polymerization. No oxidation or reduction peaks were detected in control CVs of the bare ITO-NP electrode. Using the charges under the anodic peaks, the coverages are estimated to be 1.50 × 10^−8^, 2.94 × 10^−8^, and 4.74 × 10^−8^ moles cm^−2^ for 5-, 10-, and 45-**1**-ITO-NP, respectively.

The photoredox-POP can be grown over carbon paper and bare ITO slides as well (Figure S3 in the Supplementary Materials). Figure 2c shows the reflectance Fourier transform infrared (FT-IR) spectra of both samples of the photoredox-POP films recorded after thoroughly washing the films with CH_2_Cl_2_. The peaks at about 1450 and 1610 cm^−1^ are attributed to the aromatic ring vibrations in **1** [28]. Figure S4 shows the weak signals at around 2237 cm^−1^ corresponding to the dicyano group on the central aromatic ring [43]. The strong peak at about 839 cm^−1^ is due to the hexafluorophosphate [51] counterions to the cationic nitrogen centers formed during the oxidation of the carbazole groups in **1**. The C–H stretching appears around 3000 cm^−1^ [28]. The peak around 1554 cm^−1^ is a ring vibration band characteristic of the (partially) oxidized (doped) PCz [52]. Similar observations are reported for polycarbazole prepared under the same conditions [39].

The morphology and elemental composition of 45-**1**-ITO were characterized by scanning electron microscopy (SEM) and energy dispersive X-ray spectroscopy (EDX) mapping. Figure 2d shows the edge-view SEM of a **1** film grown on an ITO slide. Figure 2e,f show the C and Sn elemental mapping, respectively. The SEM and EDX images all show a uniform distribution of the film and elements on the ITO surface.

**Figure 3:**
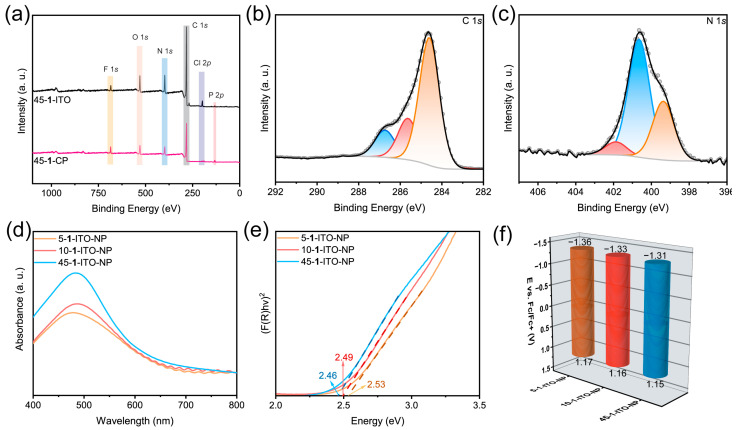
(**a**) XPS survey scans of 45-**1**-ITO and 45-**1**-CP and corresponding (**b**) C 1s and (**c**) N 1s of 45-**1**-ITO. (**d**) Reflectance UV–Vis of 5, 10, and 45-**1**-ITO-NP by reduction of the ITO-NP substrates. (**e**) Optical band gap of 5, 10, and 45-**1**-ITO-NP, and (**f**) LUMO and HOMO estimations.

Figure 3a shows the X-ray photoelectron spectroscopic (XPS) survey of both 45-**1**-ITO and 45-**1**-CP. C, N, O, P, F, and Cl are present on both photoredox-POP electrodes. The P and F signals probably arise from the presence of PF_6_^−^ counter ions to the oxidized N centers present in the polymer (Scheme 2). The Cl signal arises from residual CH_2_Cl_2_ solvent in the photoredox-POP. Figure 3b shows that deconvolution of the C 1*s* peak from 45-**1**-ITO revealed a prominent peak at 284.6 eV corresponding to sp^2^ carbons. Two additional peaks at 285.6 eV and 286.7 eV were observed and attributed to C=N and C–N (or nitrile group), respectively [53,54]. Figure 3c shows that deconvolution of the N 1*s* peak revealed three subpeaks at 399.4 eV, 400.6 eV, and 401.8 eV, which were assigned to C≡N, pyrrolic nitrogen, and imidazole nitrogen or doped (oxidized) N sites, respectively. The deconvoluted XPS of elements on 45-**1**-CP is presented in Figure S5 (see Supplementary Materials). Taken together, the CVs recorded during and after the electropolymerization, the SEM-EDX, the FTIR, and the XPS all confirmed that the electropolymerization of the imidazole-dye **1** formed the photoredox-POP shown in Scheme 2.

### 3.3. Properties of the Polymer

Figure 3d shows the reflectance UV–Vis spectra of the photoredox-POPs on ITO-NP grown over 5, 10, and 45 cycles after subtracting the ITO-NP background. The lower limit is set to 400 nm to avoid the interference of ITO [55]. The observed increase in intensity as a function of the photoredox-POP amount may be attributed to the additional growth of the conjugated repeating units of the polymer, which could give rise to a band-like distribution of energy levels within the system [56,57]. We assign this absorption to the well-known carbazole to dicyanobenzene intramolecular charge transfer [58]. The increase in wavelength of this absorption in the photoredox-POP compared with monomer **1** likely arises from either extended conjugation in the polymerized carbazole groups or perhaps some solid-state effect [59,60]. The HOMO-LUMO gaps in the three photoredox-POPs were measured by applying Kubelka–Munk theory [61]. Figure 3e shows that the HOMO and LUMO gaps were 2.53 eV (5 cycles), 2.49 eV (10 cycles), and 2.46 eV (45 cycles), respectively. As well, the energies of the LUMOs were estimated using the onset potential [62,63] for the 1 e^−^ reduction of the dicyanobenzene rings in the photoredox-POPs. Figure S6 (see Supplementary Materials) shows the CVs of the photoredox-POPs recorded in CH_2_Cl_2_. The onsets of the reduction peaks were at −1.36 V, −1.33 V, and −1.31 V vs. Fc/Fc^+^ for the 5-, 10-, and 45-cycle photoredox-POPs, respectively. Figure 3f shows the results from using the reduction onset potentials measured by CV and adding the estimated difference in voltage based on the HOMO and LUMO band gap estimated from the Kubelka–Munk plots. We note that these results are tentative and rely on many factors [64].

### 3.4. Utilization of Polymer Electrodes as Photoanodes

To investigate the photoactivity of the photoredox-POPs, we first measured the incident photon-to-current efficiencies (IPCEs) towards the photoelectrochemical oxidations of hydroquinone (HQ (0.02 M), 0.1 M NaClO_4_ in H_2_O, pH = 7.0) under neutral conditions, and of triethylamine (TEA (0.5 M), 0.1 M NaClO_4_ in H_2_O, pH = 12.6) under alkaline conditions.

**Figure 4:**
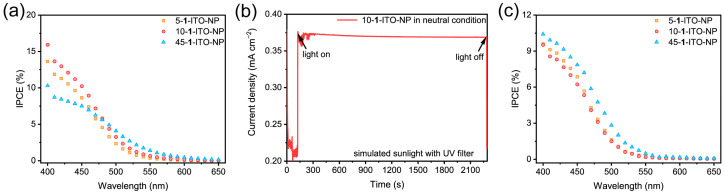
(**a**,**c**) IPCE tests of 5, 10, and 45-**1**-ITO-NP in 0.1 M NaClO_4_ dissolved in triple-distilled water containing 0.02 M hydroquinone (pH = 7.0) (neutral condition) or 0.5 M Et_3_N (pH = 12.6) (basic condition) at 0.25 V vs. SCE. (**b**) Stability test of 10-**1**-ITO-NP in the neutral condition under simulated AM 1.5G sunlight with UV-light filter.

The experiments were performed in the visible light region (400–650 nm) using a 400 nm UV-light filter. Figure 4a shows the IPCEs for the oxidation of HQ by **1** on ITO-NP deposited with 5, 10, and 45 sweeps under neutral conditions. Figure S7 (see Supplementary Materials) shows that the photocurrent of a bare ITO-NP control electrode was negligible under neutral and alkaline conditions. The photoredox-POPs were photoactive up to 550 nm (5 cycles), 560 nm (10 cycles), and 590 nm (45 cycles), showing that the activity extends further into the visible spectrum as the amount (i.e., the degree of conjugation) of the photoredox-POP increases. The maximum efficiency of all three photoredox-POPs occurs at 400 nm and decreases in the order of 15.9% (10 cycles), 13.6% (5 cycles), and 10.3% (45 cycles). These efficiencies are high for organic dyes reported in the literature in the visible light region [65,66]. It is likely that the efficiency of the 10-**1**-ITO-NP was higher than the 5-**1**-ITO-NP because of the higher number of chromophores on the surface. The efficiency of the 45-**1**-ITO-NP was slightly lower, likely because of increased resistance due to the increasing amount of photoredox-POP film. The 10-cycle electrode probably has the optimum balance between the number of chromophores per square centimeter versus electrical resistance among these three photoredox-POPs. The IPCE of the 45-cycle photoredox-POP, is, however, higher than the lower-amount films at long wavelengths, likely due to increased conjugation in the longer polymer chain. Figure 4b shows that the most active 10-**1**-ITO-NP electrode is quite stable and active under AM 1.5G sunlight. Figure S8 (see Supplementary Materials) shows that the other photoredox-POPs were appreciably stable under neutral or basic conditions.

Figure 4c shows the IPCEs of the photoredox-POPs with Et_3_N as an electron donor under basic conditions. In this case, the IPCE of the 45-**1**-ITO-NP electrode was slightly higher than the 10- or 5-**1**-ITO-NP electrodes at shorter and longer wavelengths. The maximum IPCE (~11%) and the steady-state currents were substantially higher than those we reported previously with a 4CzIPN monolayer electrografted to ITO nanoparticles using diazonium chemistry [36]. Further, chromophores attached by phosphonic- and carboxylic-acid linkers tend to decompose quickly in the presence of base. The electropolymerization of **1** occurs without derivatization, and it provides direct control over the amount of the resulting photoactive films.

### 3.5. Stilbene Isomerization by Heterogeneous Photocatalysts

**Scheme 3:**
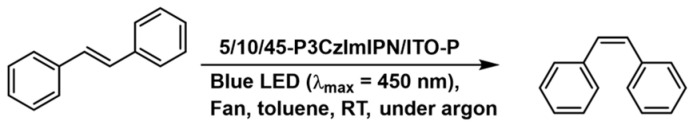
Stilbene isomerization.

We next investigated the photosynthetic isomerization of *trans-* to *cis*-stilbene using the photoredox-POPs films as heterogeneous photocatalysts (Scheme 3). These isomerizations occur via the excited state of the dye undergoing Dexter energy transfer with stilbene. The resulting triplet state of stilbene collapses to either the *trans-* or *cis-* isomer. Dexter energy transfer between the excited dye occurs preferentially with *trans*-stilbene, eventually driving the reaction towards the *cis*-isomer [40,67]. We utilized 5-**1**-ITO-NP, 10-**1**-ITO-NP, 45-**1**-ITO-NP, and ITO-NP electrodes in toluene solvent under LED irradiation at 450 nm. The amount of dye-catalyst on each photoredox-POP electrode was approximated by measuring the charge under the anodic peak for oxidation of the one-electron reduction of the dicyanobenzene groups in the CVs (Figure S9). The coverages by 5-**1**-ITO-NP, 10-**1**-ITO-NP, and 45-**1**-ITO-NP were estimated as 1.50 × 10^−8^, 2.94 × 10^−8^, and 4.74 × 10^−8^ moles cm^−2^ respectively. These values are rough estimates because other charging/background currents could not be wholly excluded. Further, these values measure all the electrochemically accessible dye centers in the photoredox-POP, not only those on the surface. Table 1 summarizes the results utilizing initial catalyst-to-stilbene ratios of 0.0060, 0.012, and 0.019, respectively. The 5-**1**-ITO-NP catalyst provided 5417.55 net turnovers from *trans* to *cis* after the first 16 h (32.49% *cis*), and 10,365.60 after 48 h (67.57% *cis*). For comparison, the 10-**1**-ITO-NP provided 1812.69 (21.34% *cis*) after 16 h and 3993.05 (52.70% *cis*) after 48 h. The ratios of turnover numbers were lower than the ratios of % *cis*, suggesting that while thicker polymers likely form with more cycles, not all of the active sites in the thicker photoredox-POPs are accessible to the reaction. The turnover numbers and % *cis* obtained with the 45-**1**-ITO-NP (949.42 after 16 h (18.01% *cis*), 2194.55 after 48 h (46.67% *cis*)) are consistent with this interpretation. The control ITO-NP electrode was essentially inactive (TON close to 0 in 48 h). We also note that the higher activity obtained with 5-**1**-ITO-NP is also consistent with the higher IPCE achieved with this electrode under neutral conditions, meaning this photoredox-POP demonstrated more effective light utilization. We next determined the photo-steady state by running a stilbene isomerization over 5-**1**-ITO-NP for a longer time. After 88 h, the conversion increased from 67.57% at 48 h to 83.33%. This photo steady state was similar to what we reported previously with a different carbazole-dicyanobenzene catalyst [36], and it resulted from the rate of the forward (cis-stilbene) and backward (trans-stilbene) reactions being the same. Reuse of the 5-**1**-ITO-NP photocatalyst resulted in a drop in efficiency by 74% at 48 h.

The internal quantum yield on a solid electrode is extremely difficult to measure accurately because other factors such as light scattering and reflections cannot be precisely determined and vary from sample to sample [68]. We determined apparent quantum efficiency based on the number of photons impinging on the solid electrode, as detailed in the Supplementary Materials. To our delight, the net external quantum yields after 16 h for 5-**1**-ITO-NP, 10-**1**-ITO-NP, and 45-**1**-ITO-NP at 450 nm were 13.73%, 9.02%, and 7.61%, respectively. These values do not include any reverse reaction that occurred during the isomerization, and so they are lower limits to the actual values. We note that these lower limits are roughly consistent with the IPCE values, indicating the inherent high performance for light utilization. These results demonstrate that the photoredox-POPs can be used as photocatalysts.

### 3.6. Photoluminescence Response to Li Ions

**Figure 5:**
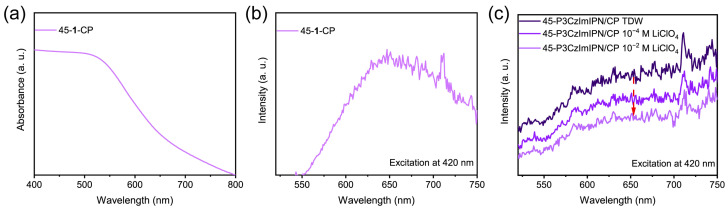
(**a**) Reflectance UV–Vis spectra and (**b**) emission when excited at 420 nm of 45-**1**-CP. (**c**) The prototype example of Li-ion detection of 45-**1**-CP.

Figure 5a shows the reflectance UV–Vis spectra of 45-**1**-CP from 400 nm to 800 nm. A thick polymer coating (45 cycles, −0.55–2.45 V vs. Fc/Fc^+^) was utilized to maximize the signal from the polymer. The background absorbance of CP was subtracted, but there still were significant absorbances from the support below ~475 nm. There was a broad peak near 510 nm that was similar to that in the spectrum of the photoredox-POPs over ITO-NP (Figure 3d). Figure 5b shows the steady-state photoluminescence spectrum. There is a broad emission peak at ~650 nm resulting from excitation at 420 nm. The corresponding emission peak for the monomer in CH_2_Cl_2_ occurs at ~550 nm. The emission from polymerized **1** on CP is broader and shifted to a longer wavelength than the monomer (Figure 1). This shift to lower energies almost certainly results from factors that include conjugation in the polymer, solid-state interactions between the polymer and the support or other polymer chains, interactions between monomers in the polymers, and solid-state packing effects on the molecular conformations. Detailed studies are required to fully understand this complex system. Regardless, the photoemission spectrum, the ICPE, and the stilbene isomerization results show that the photochemistry of the monomer is largely retained in the polymer, albeit with shifting to longer wavelengths and broadening. We also note the polycarbazole polymer has UV–Vis absorptions as well.

Considering the stability of the polymer in water and the widespread utilization of imidazole groups in sensors [52,69], we exposed the 45-**1**-CP to different concentrations of lithium in water to determine if the presence of Li affects the photoluminescence spectrum. Specifically, we soaked the 45-**1**-CP electrode in triple-distilled water (TDW) for 2 h and measured the photoluminescence spectrum. We then exposed the electrode to 10^−4^ M LiClO_4_ and 10^−2^ M LiClO_4_ aqueous solutions for 2 h. As shown in Figure 5c, the photoemissions were partially quenched by the presence of lithium, with greater quenching at higher [Li^+^]. The quenching of the photoluminescence likely originated from the coordination of Li^+^ with the imidazole groups in the photoredox-POP [70,71], suggesting that they have applications as solid-state ion sensors.

### 3.7. Understanding the Polymer

**Figure 6:**
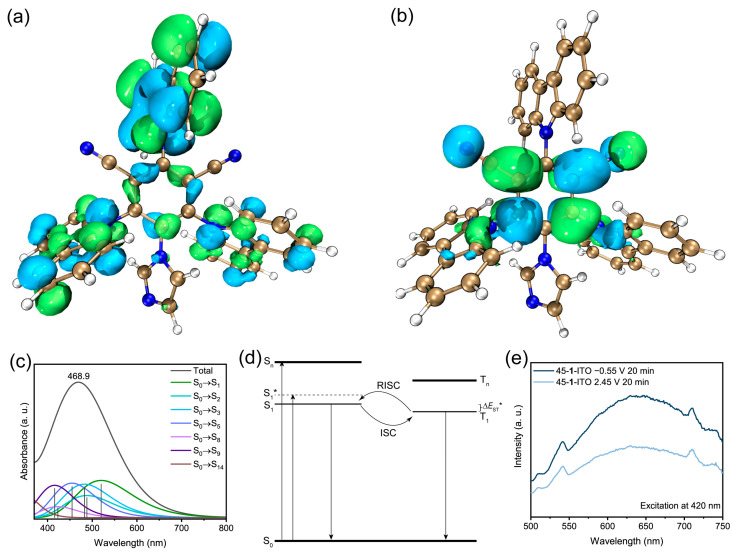
TD-DFT-calculated (**a**) HOMO and (**b**) LUMO wave functions of the geometry-optimized structures; (**c**) calculated UV–Vis spectra; (**d**), Jablonski diagram of **1**; (e) photoluminescence intensity with different applied potentials.

Preliminary time-dependent density functional theory (TD-DFT) calculations (Gaussian 16/B3LYP/6-31+G(d,p)) were carried out on **1** in the absence of solvent to provide insight into the photochemical processes reported in this paper. The calculated energy of the HOMO is −6.12 eV, and the energy of the LUMO is −3.08 eV, giving an energy gap of 3.04 eV. As shown in Figure 6a,b, the HOMO is localized on the carbazole groups, with the carbazole group between the cyano groups providing the highest contribution (LUMO, HOMO, and UV–Vis are drawn combined with Multiwfn [72] or VMD [73]). The LUMO is largely localized on the dicyanobenzene ring and roughly can be viewed as a π* aromatic orbital. As illustrated in Figure 6c, the calculated UV–Vis in the visible light region (>400 nm) is mainly a composite of six excitations. In Figure 6d, the vertical excitation energy from S_0_ to S_1_* is 2.38 eV, and the fluorescence energy from S_1_ to S_0_* is 1.84 eV (corresponding to 521 and 673.8 nm, respectively). The calculated difference in energy between S_1_ and T_1_ is small (Δ*E*_ST_*~0.18 eV), and this small difference in energy is likely one reason why intersystem crossing is relatively fast for this type of molecule [74].

Figure 6d shows the Jablonski diagram for **1**, which illustrates the potential photophysical pathways for **1**. These calculations support the results for similar compounds reported in the literature [43]. During photoexcitation, an electron is promoted from S_0_ to a higher-energy singlet state. The electron then undergoes internal conversion (IC) to reach the lowest-energy singlet excited state S_1_. Two outcomes are possible: either the electron emits fluorescent light and returns to the ground state S_0_, or it undergoes intersystem crossing (ISC) to the lowest triplet excited state T_1_. The electron in state T_1_ can either emit phosphorescent light and return to the ground state, S_0_, or undergo reverse intersystem crossing (RISC) and return to state S_1_. These calculations illustrate the photochemical processes that occur in **1** and in the photoredox-POPs.

A prediction made by combining the results from the calculations with the structure and redox functions of the polycarbazole (Scheme 2) is that the highest occupied molecular orbitals will be partially depleted at higher potentials as the carbazole groups are electro-oxidized. Depletion of these orbitals is expected to decrease the photoluminescence. Indeed, Figure 6e shows that the photoluminescence intensity of the 45-**1**-ITO photoredox-POP is significantly decreased after oxidation at 2.45 V vs. Fc/Fc^+^ for 20 min relative to the same photoredox-POP held at −0.55 V vs. Fc/Fc^+^ for 20 min. The highest occupied molecular orbitals are predominantly localized in the carbazole groups, whereas the lowest energy unoccupied orbitals primarily reside in the dicyanobenzene ring. The effect of oxidation of the carbazole groups on the light emission of the system may, for example, alter the electron transfer pathway. Specifically, upon oxidation of the carbazole groups, the excited electrons might be trapped in the holes of the oxidized carbazole groups. Regardless of the exact mechanism, these results show that the photoluminescence behavior of these photoredox-POPs can to some extent be modulated by the applied potential.

## 4. Conclusions

A one-step preparation of the imidazole-functionalized, polycarbazole dicyanobenzene dye **1** is reported. The photophysical behavior of **1** is typical of this class of organic chromophores [10]. The dye is readily electropolymerized in air at the carbazole rings to produce photoredox-POPs on ITO glass, nanoparticles, and carbon paper. The resulting deposits are active photoanodes, photocatalysts for olefin isomerization reactions, and their photoluminescence behavior responds to lithium ions in solution, presumably through the coordinating imidazole groups. Studies are underway in our laboratories to fully investigate the photochemical properties and utility of these new photoredox-POP deposits.

## Figures and Tables

**Table 1:** Photosynthetic isomerization of *trans*- to *cis*-stilbene with different ITO-NP electrodes.

Catalyst ^1^	Mol % of Catalyst	[Stilbene]/M	%conv (16 h)	TON (16 h)	TOF/h (16 h)	%conv (48 h)	TON (48 h)	TOF/h (48 h)	%conv (88 h)
5-**1**-ITO-NP	0.0060	0.1	32.49	5417.55	338.60	67.57	10,365.60	215.95	
10-**1**-ITO-NP	0.012	0.1	21.34	1812.69	113.29	52.70	3993.05	83.19	
45-**1**-ITO-NP	0.019	0.1	18.01	949.42	59.34	46.67	2194.55	45.72	
ITO-NP	0	0.1	~0	~0	~0	~0	~0	~0	
5-**1**-ITO-NP ^2^	0.0060	0.1							83.33
5-**1**-ITO-NP ^3^		0.1	4	666.98	41.69	17.63	2704.537	56.34	

^1^ In the absence of any explicit notation to the contrary, the isomerization reactions were conducted using *trans*-stilbene as the substrate, toluene as the solvent, and an argon atmosphere at ambient temperature, with various cycles of photoredox-POPs serving as the catalyst. The % conversion, TON (with TONs being calculated after subtracting the amount of substrate removed in the preceding aliquots), and TOFs were determined through ^1^H NMR spectroscopy. ^2^ New run of a new 5-**1**-ITO-P electrode. ^3^ Second run of the same 5-**1**-ITO-P electrode reused.

## Data Availability

The data presented in this study are available upon reasonable request from the corresponding author.

## Supplementary Materials

The following supporting information can be downloaded at: https://www.mdpi.com/article/10.3390/polym15163340/s1, Figure S1. (a) CV curves of 5-**1**-ITO-NP in 0.1 M TBAPF_6_ and 1 mM **1** in DCM. (b) CV curves of 45-**1**-ITO-NP in 0.1 M TBAPF_6_ and 1 mM **1.** Sweep rate 100 mV s^−1^, 5 or 45 sweeps, sweep range from −0.55 to 1.45 V vs. Fc/Fc^+^. (c) Determination of E_1/2_ for Fc/Fc^+^ reference in the same solution; Figure S2. (a) Negative region CV curves for isolated 5-, 10-, and 45-**1**-ITO-NP compared to an ITO-NP blank showing the 1 e^−^ redox wave for the dicyanobenzene groups in the polymers (0.1 M TBAPF_6_ in MeCN, sweep rate 10 mV s^−1^ from −0.41 to −1.91 V vs. Fc/Fc^+^); (b) determination of E_1/2_ for Fc/Fc^+^ reference in the same solution; Figure S3. (a) CV curves for electropolymerization of **1** to prepare 45-**1**-ITO and (b) 45-**1**-CP in 0.1 M TBAPF_6_ and 1 mM **1** in DCM (45 sweeps, sweep rate 100 mV s^−1^ from −0.55 to 2.45 V vs. Fc/Fc^+^); Figure S4. Enlarged FT-IR region with putative assignments of the weak stretching peaks for the dicyano groups on the central aromatic ring in 45-**1**-ITO and 45-**1**-CP; Figure S5. The deconvoluted XPS of elements (a) C 1*s*, (b) N 1*s*, and (c) O 1*s* on 45-**1**-CP; Figure S6. CVs (sweep rate 10 mV s^−1^ from −0.30 to −1.86 V vs. Fc/Fc^+^) of the 5, 10, or 45-**1**-ITO-NP electrodes immersed in CH_2_Cl_2_ with 0.1 M TABPF_6_. The onset potentials were determined as shown in the figure; Figure S7. IPCE measurements with the ITO-NP control, and (a) 10-**1**-ITO-NP electrodes under neutral and (b) 45-**1**-ITO-NP electrodes under alkaline conditions; 0.02 M hydroquinone (pH = 7.0) (neutral condition) or 0.5 M Et_3_N (pH = 12.6) (basic condition) in triple-distilled water at 0.25 V vs. SCE. Each spike in current corresponds to a decrease in wavelength by 10 nm, starting at 700 nm, then down to 400 nm. The light was held at each wavelength for ~8 s; Figure S8. (a) The steady-state potentiostatic photoelectro-oxidation of hydroquinone over 10-**1**-ITO-NP under neutral conditions, and of triethyl amine over 45-**1**-ITO-NP under basic conditions; 0.02 M hydroquinone (pH = 7.0) (neutral condition) or 0.5 M Et_3_N (pH = 12.6) (basic condition) in triple-distilled water at 0.25 V vs. SCE; Figure S9. Estimation of electrochemically accessible surface coverage of poly **1** from the charge under the one-electron redox wave for the dicyanobenzene moieties in the polymer. 5-**1**-ITO-NP: 1.50 × 10^−8^ mol; 10-**1**-ITO-NP: 2.94 × 10^−8^ mol; 45-**1**-ITO-NP: 4.74 × 10^−8^ mol; Figure S10. Working diagram of the synthesis procedures for ploy-3CzImIPN (**1**); Figure S11. (a) SEM image of the surface of the 45-**1**-ITO film. This image was recorded without protecting the surface with layers of tungsten and gold. (b) SEM cross-section view to measure the polymer thickness of 45-**1**-ITO. The polymer was protected with a layer of tungsten and gold before this measurement was recorded; Figure S12. ^1^H NMR of 3CzImIPN (**1**). The δ region from 10 to 1 ppm showing ^1^H NMR. The spectrum was acquired in CD_3_CN with a 500 MHz Varian Inova; Figure S13. Magnified ^1^H NMR of 3CzImIPN (**1**). The δ region from 9 to 6 ppm showing ^1^H NMR. The spectrum was acquired in CD_3_CN with a 500 MHz Varian Inova; Figure S14. ^13^C NMR of 3CzImIPN (**1**) from 0 to 190 ppm. The spectrum was acquired in acetone with a Varian VNMRS 500 MHz NMR spectrometer; Figure S15. Magnified ^13^C NMR of 3CzImIPN (**1**) from 100 to 160 ppm. The spectrum is acquired in acetone by Varian VNMRS 500 MHz NMR spectrometer; Figure S16. Mass spectrometry of 3CzFIPN; Figure S17. Mass spectrometry of 3CzImIPN (**1**); Figure S18. ^1^H NMR of stilbene isomerization by ITO-NP for 16 h. The δ region from 10 to 0 ppm; Figure S19. ^1^H NMR of stilbene isomerization by ITO-NP for 48 h. The δ region from 10 to 0 ppm; Figure S20. Magnified ^1^H NMR of stilbene isomerization by new ITO-NP for 48 h. The δ region from 6 to 8 ppm showing ^1^H NMR; Figure S21. ^1^H NMR of stilbene isomerization by 5-**1**-ITO-NP for 16 h. The δ region from 10 to 0 ppm showing ^1^H NMR; Figure S22. ^1^H NMR of stilbene isomerization by 5-**1**-ITO-NP for 48 h. The δ region from 10 to 0 ppm showing ^1^H NMR; Figure S23. Magnified ^1^H NMR of stilbene isomerization by 5-**1**-ITO-NP for 48 h. The δ region from 6 to 8 ppm showing ^1^H NMR; Figure S24. ^1^H NMR of stilbene isomerization by 10-**1**-ITO-NP for 16 h. The δ region from 10 to 0 ppm showing ^1^H NMR; Figure S25. ^1^H NMR of stilbene isomerization by 10-**1**-ITO-NP for 48 h. The δ region from 10 to 0 ppm showing ^1^H NMR; Figure S26. Magnified ^1^H NMR of stilbene isomerization by 10-**1**-ITO-NP for 48 h. The δ region from 6 to 8 ppm showing ^1^H NMR; Figure S27. ^1^H NMR of stilbene isomerization by 45-**1**-ITO-NP for 16 h. The δ region from 10 to 0 ppm showing ^1^H NMR; Figure S28. ^1^H NMR of stilbene isomerization by 45-**1**-ITO-NP for 48 h. The δ region from 10 to 0 ppm showing ^1^H NMR; Figure S29. Magnified ^1^H NMR of stilbene isomerization by 45-**1**-ITO-NP for 48 h. The δ region from 6 to 8 ppm showing ^1^H NMR; Figure S30. ^1^H NMR of stilbene isomerization by the second run of 5-**1**-ITO-NP for 16 h. The δ region from 10 to 0 ppm showing ^1^H NMR; Figure S31. ^1^H NMR of stilbene isomerization by the second run of 5-**1**-ITO-NP for 48 h. The δ region from 10 to 0 ppm showing ^1^H NMR; Figure S32. Magnified ^1^H NMR of stilbene isomerization by the second run of 5-**1**-ITO-NP for 48 h. The δ region from 6 to 8 ppm showing ^1^H NMR; Figure S33. ^1^H NMR of stilbene isomerization by new 5-**1**-ITO-NP for 88 h. The δ region from 10 to 0 ppm showing ^1^H NMR; Figure S34. Magnified ^1^H NMR of stilbene isomerization by 5-**1**-ITO-NP for 88 h. The δ region from 6 to 8 ppm showing ^1^H NMR; Table S1. Crystallographic experimental details. References [36,75,76,77,78] are cited in the Supplementary Materials.
