# Supplementary material for: One-Step Electropolymerization of a Dicyanobenzene-Carbazole-Imidazole Dye to Prepare Photoactive Redox Polymer Films
Source: Polymers (Basel). 2023 Aug 8;15(16):3340. doi: 10.3390/polym15163340 (PMC10457835; doi:10.3390/polym15163340)
Supplement: Supplementary file 1 [file polymers-15-03340-s001.zip › polymers-2521590-supplementary.pdf]

Supplementary Materials

Article

One-Step Electropolymerization of a Dicyanobenzene-Carbazole-Imidazole Dye to Prepare Photoactive Redox Polymer Films

Jinkun Liu [1], Octavio Martinez Perez [1], Dominic Lavergne [1], Loorthuraja Rasu [1], Elizabeth Murphy [1], Andy Galvez-Rodriguez [1] and Steven H. Bergens [1,*]

Department of Chemistry, University of Alberta, 11227 Saskatchewan Drive, Edmonton, Alberta T6G 2G2, Canada

* Correspondence: sbergens@ualberta.ca

Supporting Information Table of Contents

General Information S3

Supplementary Figures S5

Synthesis of Target Compounds S10

Supplementary NMR Spectra S17

Crystallographic Experimental Details for 3CzImIPN (1) S34

Supplementary References S35

General Information

Photoelectrochemistry. The photoactivity of the electrodes was investigated in two different pH solutions: a 0.1 M $NaClO_4$ water solution containing 0.02 M hydroquinone (pH = 7.0), and a 0.1 M $NaClO_4$ water solution containing 0.5 M triethylamine (pH = 12.6). The reference and counter electrodes were saturated calomel electrode (SCE) and high surface area platinum gauze, respectively. The reaction was carried out in a four-neck quartz flask (total volume of 100 mL) with a flat window used as the reaction flask. The electrolyte was stirred using a magnetic bar during the measurements. Before each experiment, the electrolyte was purged with argon for 20 min, and an argon gas atmosphere was maintained on top of the solution throughout the studies. The light source was a 300 W Xe lamp equipped with a Cornerstone M 260 QEPVSI-b monochromator from Newport. The incident light intensity was determined using a Thorlab S121C light meter at each wavelength. The incident photon-to-current conversion efficiencies (IPCEs) at any given wavelength was calculated using Equation (1):

$$IPCE\ (\%) = (1240\ i/(P\ \lambda)) \times 100 \qquad (1)$$

where λ is the specific wavelength of irradiated light (nm), P is light power density (mW cm^{-2}), and i is the measured photocurrent density (mA cm^{-2}). For electrochemical studies, a Solartron SI 1287 potentiostat equipped with an SI 1260 frequency response analyzer was used.

Estimation of surface coverage on the electrodes using cyclic voltammetry (CV). Initially, the I–t curves were generated based on the corresponding CV curves with known scan rates. The total charge in Coulombs (Q) was subsequently calculated by integrating the dicyanobenzene oxidation peak. Additionally, the number of moles of the substance on the electrodes (mole) was determined using Equation (2) as follows:

$$mole = Q/(N_A\ e) \qquad (2)$$

where Q is the total charge in Coulombs, N_A is the Avogadro constant, and e is the Coulomb number for one electron.

Estimation of Film Transparency. To calculate the transparency value, we measured the light transmittance at 600 nm using a monochromator and employed Equation (3) as follows [75]:

$$Transparency = -\log T600/x \qquad (3)$$

where $T600$ is the fractional transmittance at 600 nm, and x is the film thickness (mm). We initially measured the light intensity unobstructed (noted as 100%). Subsequently, with an ITO glass placed in front of the detector, the light intensity decreased to 85.7% compared to the unobstructed light intensity. The fractional transmittance of the polymer film was determined by placing 45-**1**-ITO in front of the detector at the same position as the ITO glass. The transmitted light intensity decreased to 80.0% compared to the unobstructed light intensity. Thus, the estimated fractional transmittance at 600 nm for polymer film was calculated as 100% – (85.7% – 80.0%) = 94.3%.

To ascertain the thickness of the polymer film, we conducted a cross-sectional view of SEM (Figure S11). Lastly, the film transparency of the polymer film was computed using Equation (3) as 70.4. Note that there are many factors of polymer film on ITO glass, such as diffuse reflection between the polymer and the ITO layer.

Determination of apparent quantum efficiency.

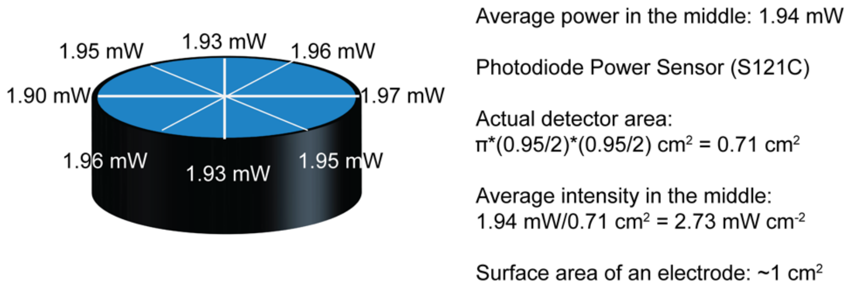

The reactor apparatus was prepared with either a **1**-ITO-NP glass slide electrode or an ITO-NP glass slide electrode in a glass vial, which was filled with a solution of *trans*-stilbene at the start of the experiment. To ensure an argon atmosphere, the vial containing the sample was transferred to a larger Schlenk line side-arm flask, which was then positioned at the center of a glass cylinder wrapped with LED tape emitting at 450 nm. The intensity of the light was measured at 45° intervals, with a photodiode power sensor positioned at the center of the vial. The average intensity in the middle of the vial was found to be 2.73 mW cm^{-2}, and the surface area of one electrode was approximately 1 cm^2. Based on these values, the total energy of the incident photon flux over 16 h was calculated as 157.25 J using the formula 1.94×10^{-3} (J s^{-1}) (cm^{-2}) × 1 cm^2 × (16 × 60 × 60) s. This result corresponds to 0.59 mmol photons (by 157.25 J/ 4.41424×10^{-19} J /N_A; the energy of one photon at 450 nm is 4.41424×10^{-19} J; N_A is Avogadro's constant). Apparent quantum efficiency (AQE) = # of moles of *cis*-stilbene produced/# of moles of photons AQY = 0.25 mmol x 32.49%/0.59154 mmol = 13.73% (5 cycles); 9.02% (10 cycles); 7.61% (45 cycles).

Calculation Method. We employed the Gaussian 16 software [76] package to conduct the Time-Dependant Density Functional Theory (TD-DFT) calculations. Specifically, the molecular structures were computed using the B3LYP (Becke, three-parameter, Lee–Yang–Parr) hybrid functional [77] to describe the exchange–correlation energies. Basis sets were implemented to enable high-level B3LYP calculations at 6-31+G(d,p) [78] for atoms, including C, N, and H. Vibrational frequency calculations were performed on all minima states, and each minimum was identified to have no imaginary frequencies. Prior to energy-related calculations, the ground state was stabilized to the minimum energy point. Notably, all excited-state energies were found to be positive during the calculation process. To determine the T1 energy, we first optimized it using the Unrestricted Kohn–Sham (UKS) method. Additionally, LUMO and HOMO were drawn using Gaussian 16, Multiwfn (Multiwfn_3.8_dev_bin_Win64), and VMD (1.9.3). Furthermore, the UV–Vis was calculated via Gaussian 16 (n-states = 80) and was subsequently read and exported by Multiwfn (Multiwfn_3.8_dev_bin_Win64).

Supplementary Figures

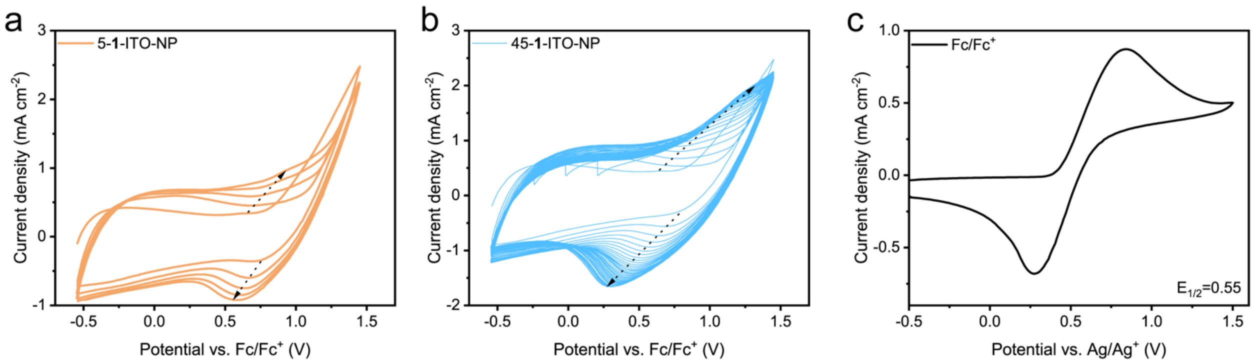

Figure S1. (a) CV curves of 5-**1**-ITO-NP in 0.1 M $TBAPF_6$ and 1 mM **1** in DCM. (b) CV curves of 45-**1**-ITO-NP in 0.1 M $TBAPF_6$ and 1 mM **1.** Sweep rate 100 mV s^{-1}, 5 or 45 sweeps, sweep range from -0.55 to 1.45 vs. Fc/Fc^+. **(**c) Determination of $E_{1/2}$ for Fc/Fc^+ reference in the same solution.

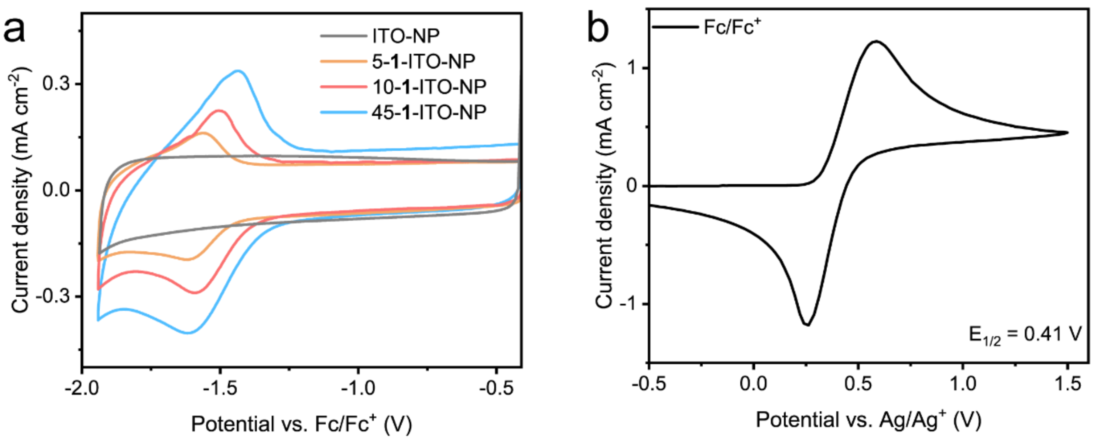

Figure S2. (a) Negative region CV curves for isolated 5-, 10-, and 45-**1**-ITO-NP compared to an ITO-NP blank showing the 1 e^- redox wave for the dicyanobenzene groups in the polymers (0.1 M $TBAPF_6$ in MeCN, sweep rate 10 mV s^{-1} from -0.41 to -1.91 vs. Fc/Fc^+); (b) determination of $E_{1/2}$ for Fc/Fc^+ reference in the same solution.

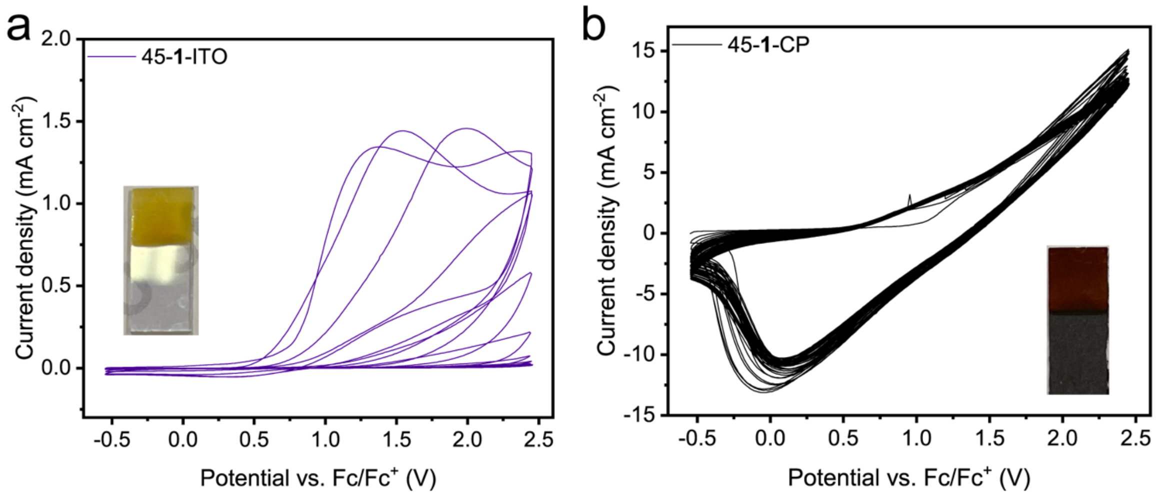

Figure S3. (a) CV curves for electropolymerization of **1** to prepare 45-**1**-ITO and (b) 45-**1**-CP in 0.1 M $TBAPF_6$ and 1mM **1** in DCM (45 sweeps, sweep rate 100 mV s^{-1} from -0.55 to 2.45 vs. Fc/Fc$^+$).

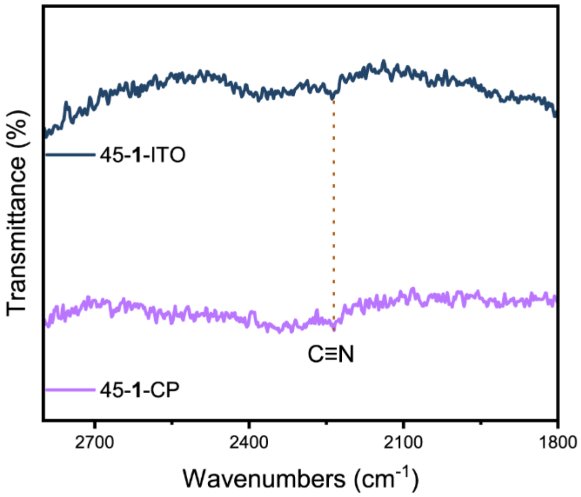

Figure S4. Enlarged FT-IR region with putative assignments of the weak stretching peaks for the dicyano groups on the central aromatic ring in 45-**1**-ITO and 45-**1**-CP.

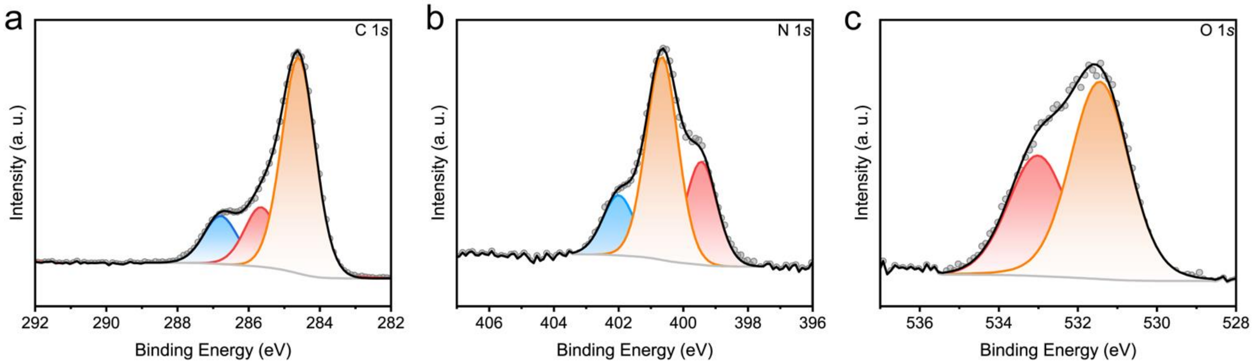

Figure S5. The deconvoluted XPS of elements (a) C 1*s*, (b) N 1*s*, and (c) O 1*s* on 45-**1**-CP.

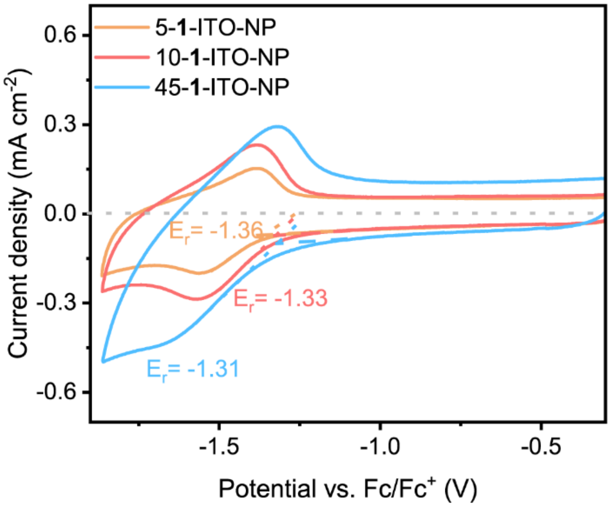

Figure S6. CVs (sweep rate 10 mV s^{-1} from -0.30 to -1.86 vs. Fc/Fc^{+}) of the 5, 10, or 45-**1**-ITO-NP electrodes immersed in CH_2Cl_2 with 0.1 M $TABPF_6$. The onset potentials were determined as shown in the figure.

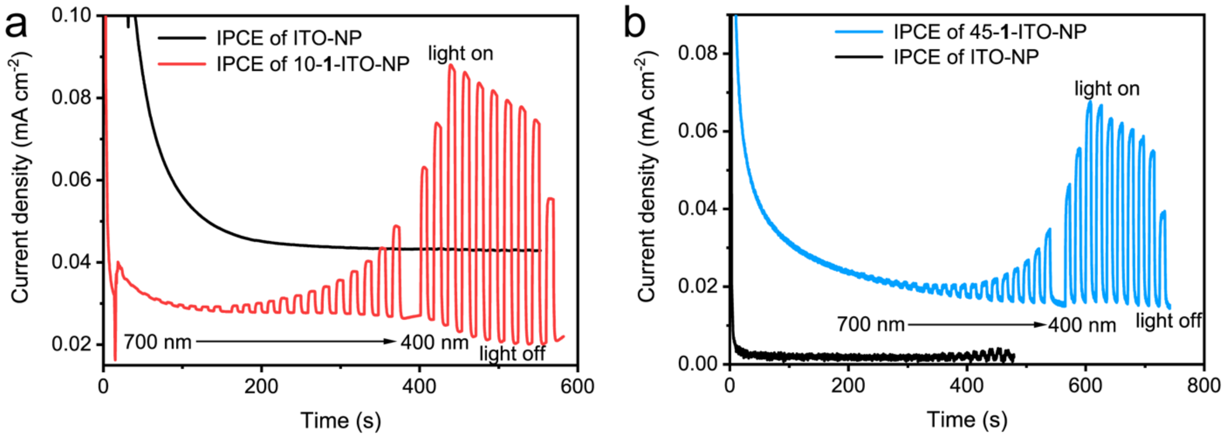

Figure S7. IPCE measurements with the ITO-NP control, and (a) 10-**1**-ITO-NP electrodes under neutral and (b) 45-**1**-ITO-NP electrodes under alkaline conditions; 0.02 M hydroquinone (pH = 7.0) (neutral condition) or 0.5 M Et_3N (pH = 12.6) (basic condition) in triple-distilled water at 0.25 V vs. SCE. Each spike in current corresponds to a decrease in wavelength by 10 nm, starting at 700 nm, then down to 400 nm. The light was held at each wavelength for ~8 seconds.

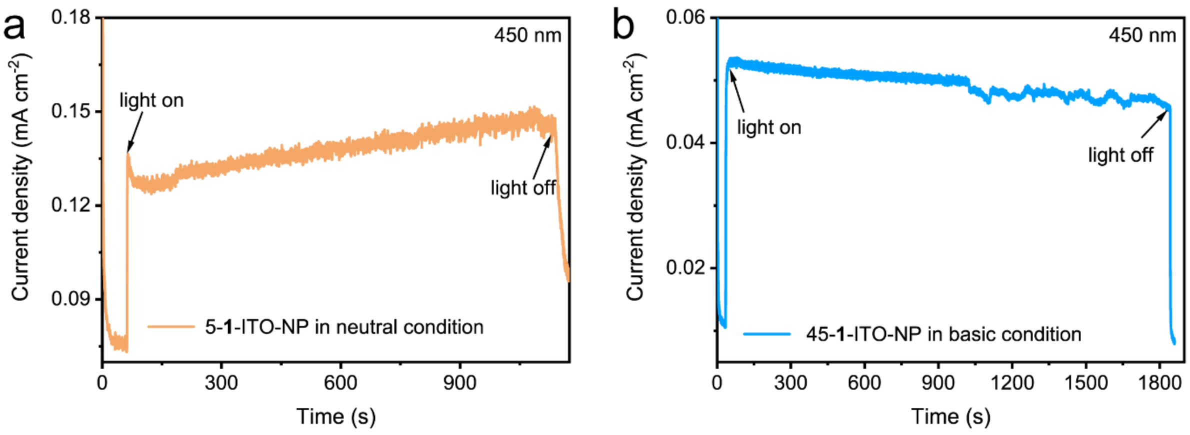

Figure S8. (a) The steady-state potentiostatic photoelectro-oxidation of hydroquinone over 10-1-ITO-NP under neutral conditions, and of triethyl amine over 45-1-ITO-NP under basic conditions; 0.02 M hydroquinone (pH = 7.0) (neutral condition) or 0.5 M Et_3N (pH = 12.6) (basic condition) in triple-distilled water at 0.25 V vs. SCE.

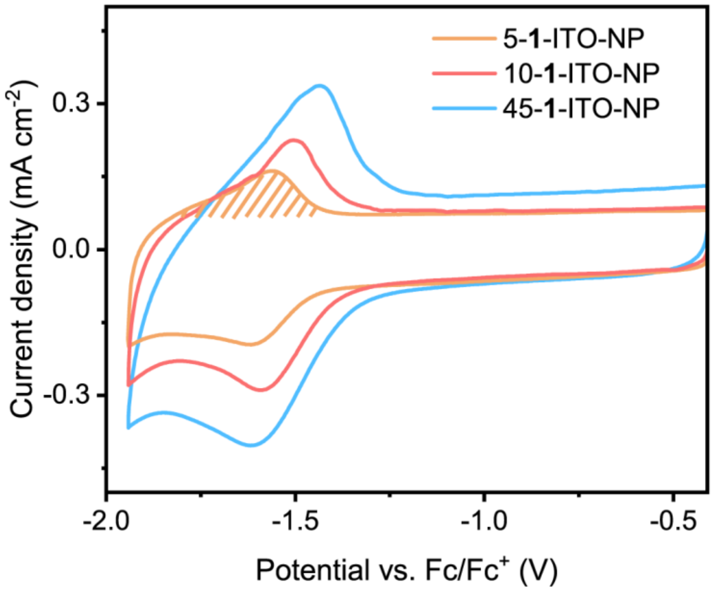

Figure S9. Estimation of electrochemically accessible surface coverage of poly **1** from the charge under the one-electron redox wave for the dicyanobenzene moieties in the polymer. 5-**1**-ITO-NP: 1.50 x 10^{-8} mol; 10-**1**-ITO-NP: 2.94 x 10^{-8} mol; 45-**1**-ITO-NP: 4.74 x 10^{-8} mol.

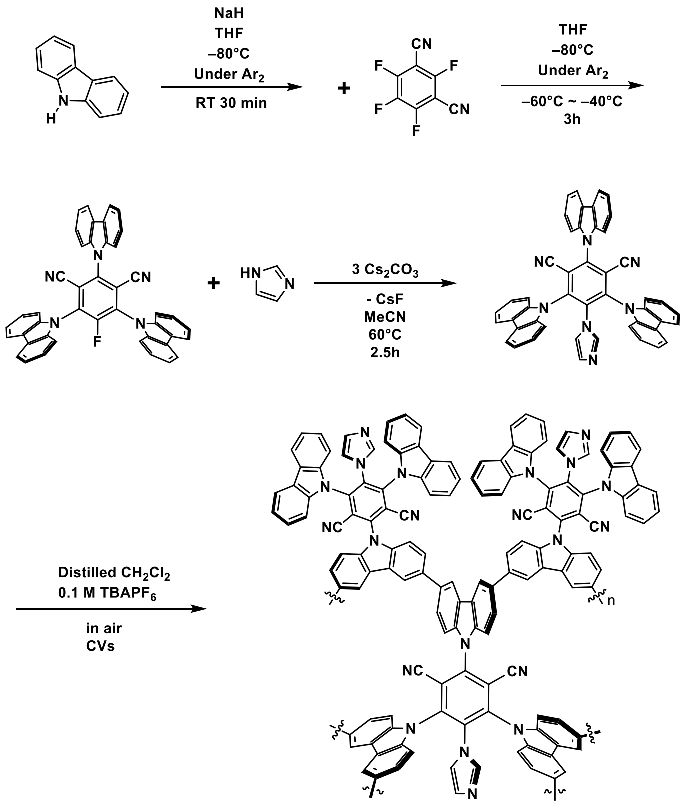

Figure S10. Working diagram of the synthesis procedures for poly-3CzImIPN (**1**).

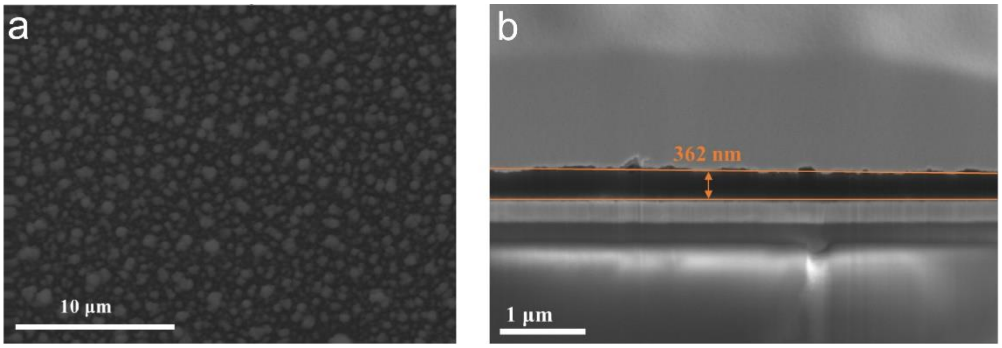

Figure S11. (a) SEM image of the surface of the 45-**1**-ITO film. This image was recorded without protecting the surface with layers of tungsten and gold. (b) SEM cross-section view to measure the polymer thickness of 45-**1**-ITO. The polymer was protected with a layer of tungsten and gold before this measurement was recorded.

Synthesis of Target Compounds

5-fluoro-2,4,6-tri(9H-carbazol-9-yl)benzene-1,3-dicarbonitrile (3CzFIPN) was prepared as described in our previous report [36].

1-imidazole-2,4,6-tri(carbazol-9-yl)-3,5-dicyanobenzene (3CzImIPN) (1). Imidazole (106.1 mg, 1.56 mmol) and Cs_2CO_3 (1.52 g, 4.67 mmol) were weighed into a side-arm flask. The flask was then connected to a Schlenk line, evacuated, and refilled with argon for 3 cycles. Acetonitrile (CH_3CN, 30 mL, dried over molecular sieves and bubbled with N_2 for 30 minutes) was then added into the flask, and the resulted suspension was stirred at 50 °C for 30 minutes. Then, the monofluro compound, 3CzFIPN (1.06 g, 1.56 mmol) was dissolved in 10 mL of CH_3CN and added slowly into the flask containing imidazole and base at room temperature. Additional CH_3CN (10 mL) was used for quantitative transfer of 3CzFIPN, and then the contents in the flask were stirred at 50 °C. An aliquot after three hours showed the completion of the reaction with no starting materials remaining. The reaction flask was cooled down and filtered with an additional wash with CH_3CN (100 mL). The combined filtrate was then concentrated under reduced pressure using a rotavap to yield yellow solid products. The crude product was then dissolved in DCM (50 mL) and washed with water (3 × 50 mL). After washing with saturated NaCl, the organic layer was dried over anhydrous Na_2SO_4, filtered, and concentrated again using a rotavap to yield the target product as a yellow powder in 95% yield. **^{1}H NMR** (499.789 MHz, CD_3CN, 27.7 °C): δ 6.11 (1H, s), 6.21 (1H, t, J = 1.3 Hz), 6.73 (1H, s), 7.39 (4H, t, J = 7.5 Hz), 7.49–7.55 (6H, m), 7.62 (4H, d, J = 8.1), 7.73 (1H, dt, J = 8.0 Hz, J = 1.1 Hz), 7.86 (1H, d, J = 8.2 Hz), 8.17 (2H, d, J = 7.8 Hz), 8.30 (2H, d, J = 7.8 Hz). **^{13}C{^{1}H} NMR** (125.685 MHz, CD3CN, 27.7 °C): δ 110.1, 110.6, 111.8, 117.8, 118.2, 120.8, 121.0, 122.1, 122.4, 123.9, 124.2, 126.9, 127.1, 129.3, 135.4, 137.3, 139.4, 139.7, 143.2, 145.3. **HRMS (ESI)** m/z Calcd. for $C_{47}H_{27}N_7$ $(M+H)^+$: 690.2401. Found: 690.2404.

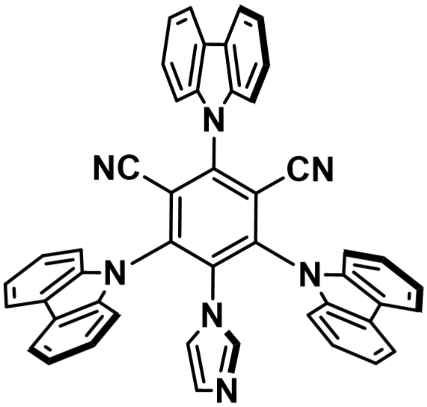

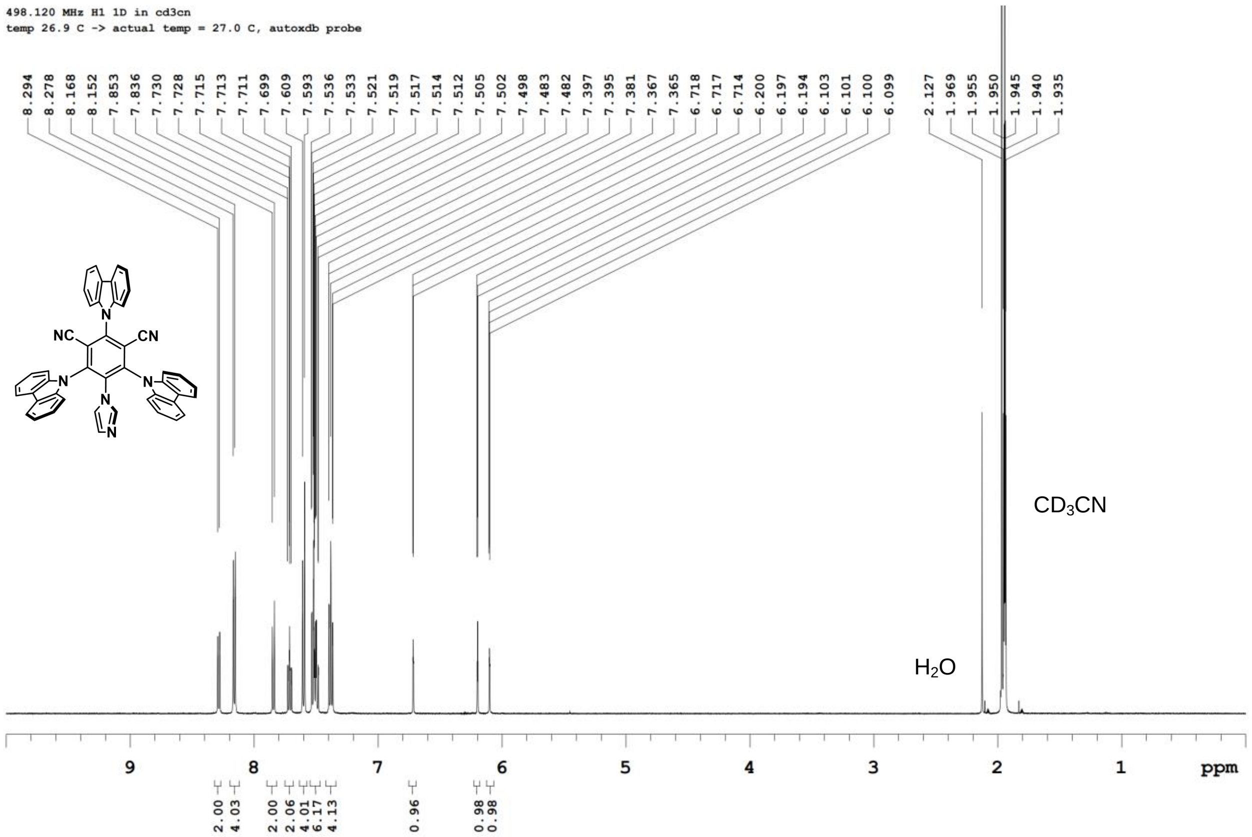

Figure S12. ^{1}H NMR of 3CzImIPN (**1**). The δ region from 10 to 1 ppm showing ^{1}H NMR. The spectrum was acquired in CD_3CN with a 500 MHz Varian Inova.

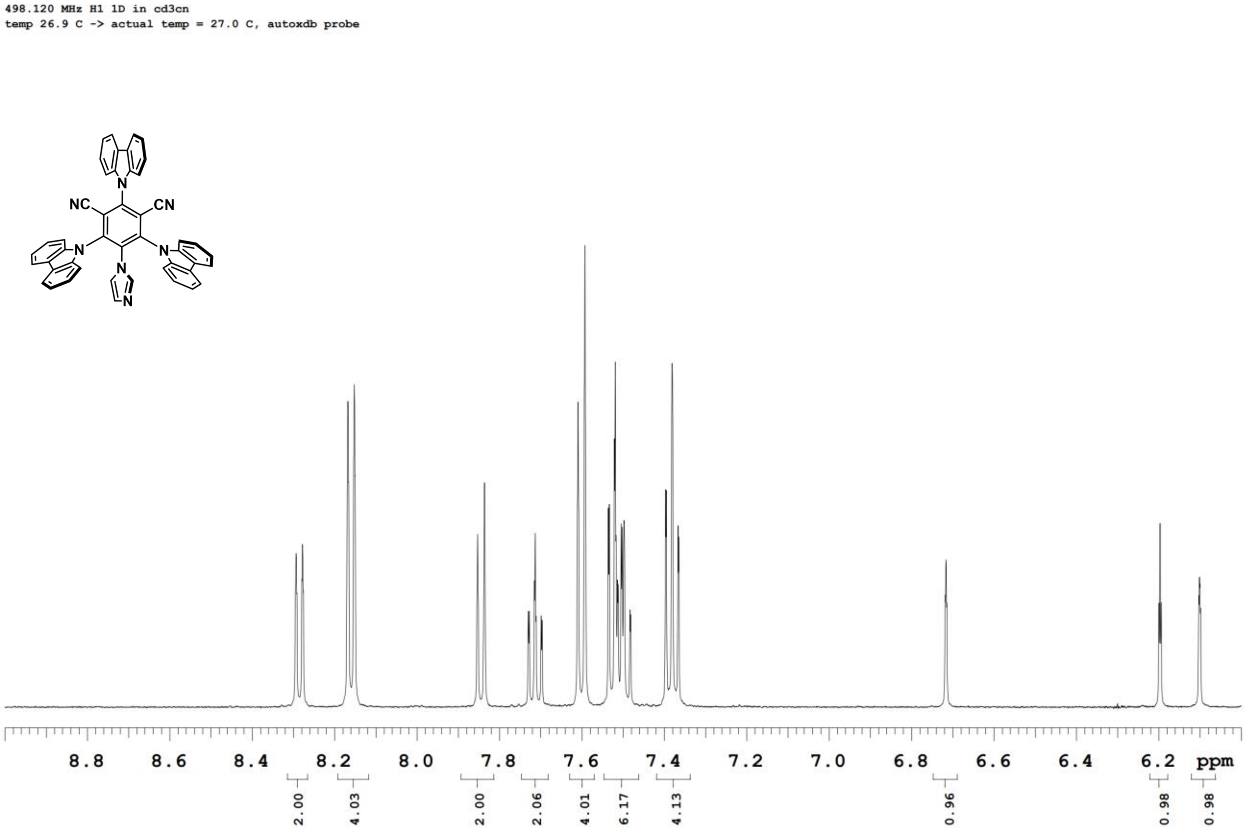

Figure S13. Magnified ^{1}H NMR of 3CzImIPN (**1**). The δ region from 9 to 6 ppm showing ^{1}H NMR. The spectrum was acquired in CD_3CN with a 500 MHz Varian Inova.

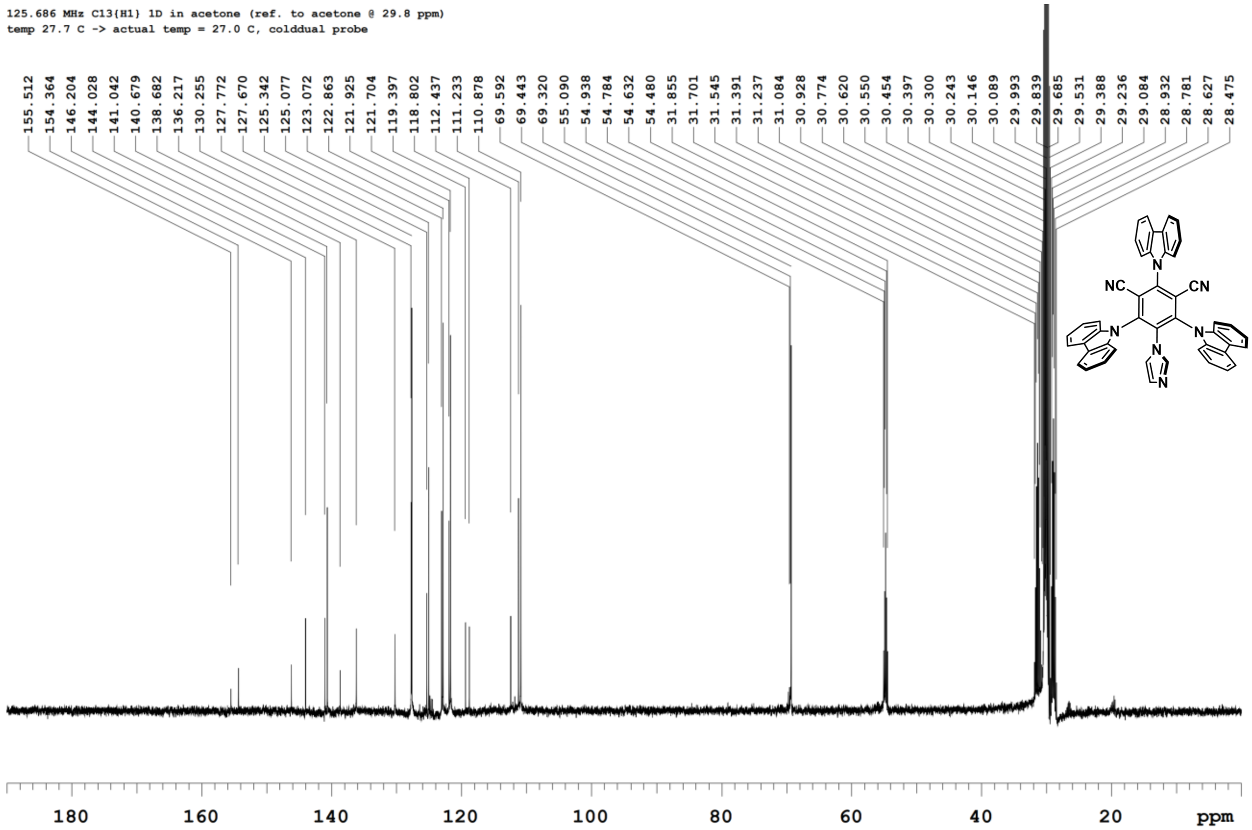

Figure S14. ^{13}C NMR of 3CzImIPN (**1**), from 0 to 190 ppm. The spectrum was acquired in acetone using a Varian VNMRS 500 MHz NMR spectrometer.

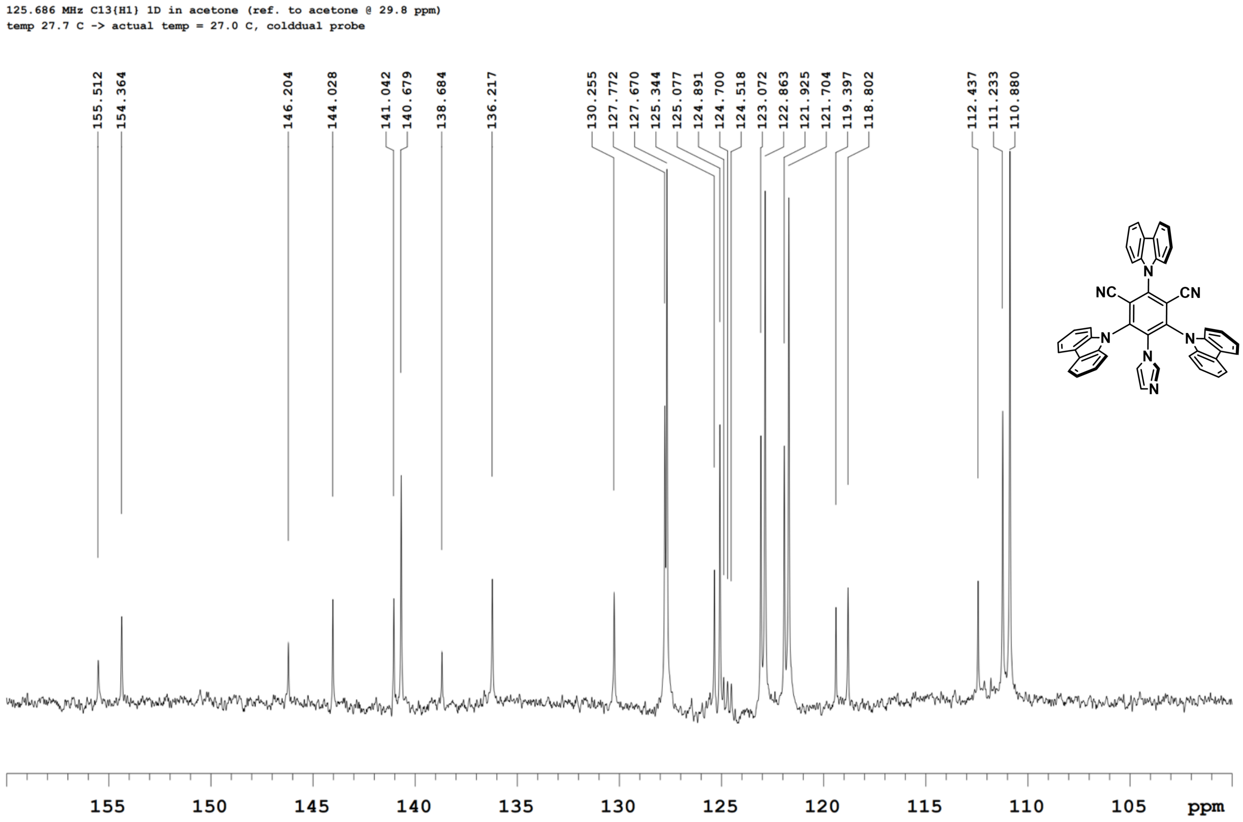

Figure S15. Magnified ^{13}C NMR of 3CzImIPN (**1**), from 100 to 160 ppm. The spectrum was acquired in acetone using a Varian VNMRS 500 MHz NMR spectrometer.

Qualitative Analysis Report

**Data Filename**	23071906.d	**Name**	O. Martinez Perez, Bergens
**Sample Name**	c44h24fn5	**Position**	-1
**Instrument Name**	oaTOF6220	**Operator**	ami
**Acq Method**		**DA Method**	ami_da.m

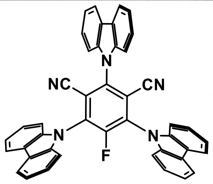

User Spectra

Fragmentor Voltage	Collision Energy	Ionization Mode
250	0	Esi

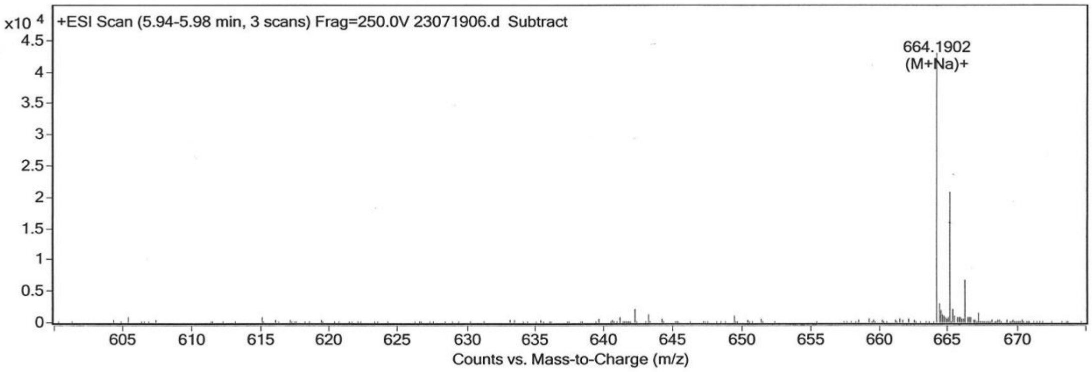

Formula Calculator Results

Formula	Ion Formula	Mass	Calc Mass	m/z	Calc. m/z	Diff (mDa)	Diff (ppm)	DBE	Ion Species	Score
C44 H24 F N5	C44 H24 F N5 Na	641.2009	641.2016	664.1902	664.1908	0.62	0.93	35	(M+Na)+	93.65

--- End Of Report ---

Figure S16. Mass spectrometry of 3CzFIPN.

Qualitative Analysis Report

**Data Filename**	23071905.d	**Name**	O. Martinez Perez, Bergens
**Sample Name**	c47h27n7	**Position**	-1
**Instrument Name**	oaTOF6220	**Operator**	ami
**Acq Method**		**DA Method**	ami_da.m

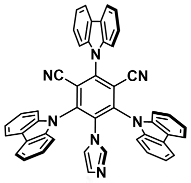

User Spectra

Fragmentor Voltage	Collision Energy	Ionization Mode
200	0	Esi

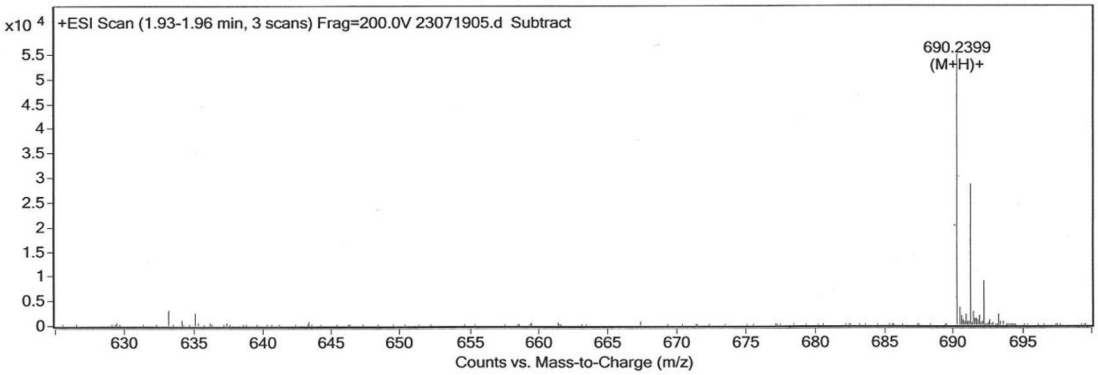

Formula Calculator Results

Formula	Ion Formula	Mass	Calc Mass	m/z	Calc. m/z	Diff (mDa)	Diff (ppm)	DBE	Ion Species	Score
C47 H27 N7	C47 H28 N7	689.2326	689.2328	690.2399	690.2401	0.13	0.19	38	(M+H)+	94.25

--- End Of Report ---

Figure S17. Mass spectrometry of 3CzImIPN (**1**).

Supplementary NMR Spectra

Stilbene Isomerization. *Trans*-reactants denoted by (T); *cis*-products denoted by (*); internal standard denoted by (IS)

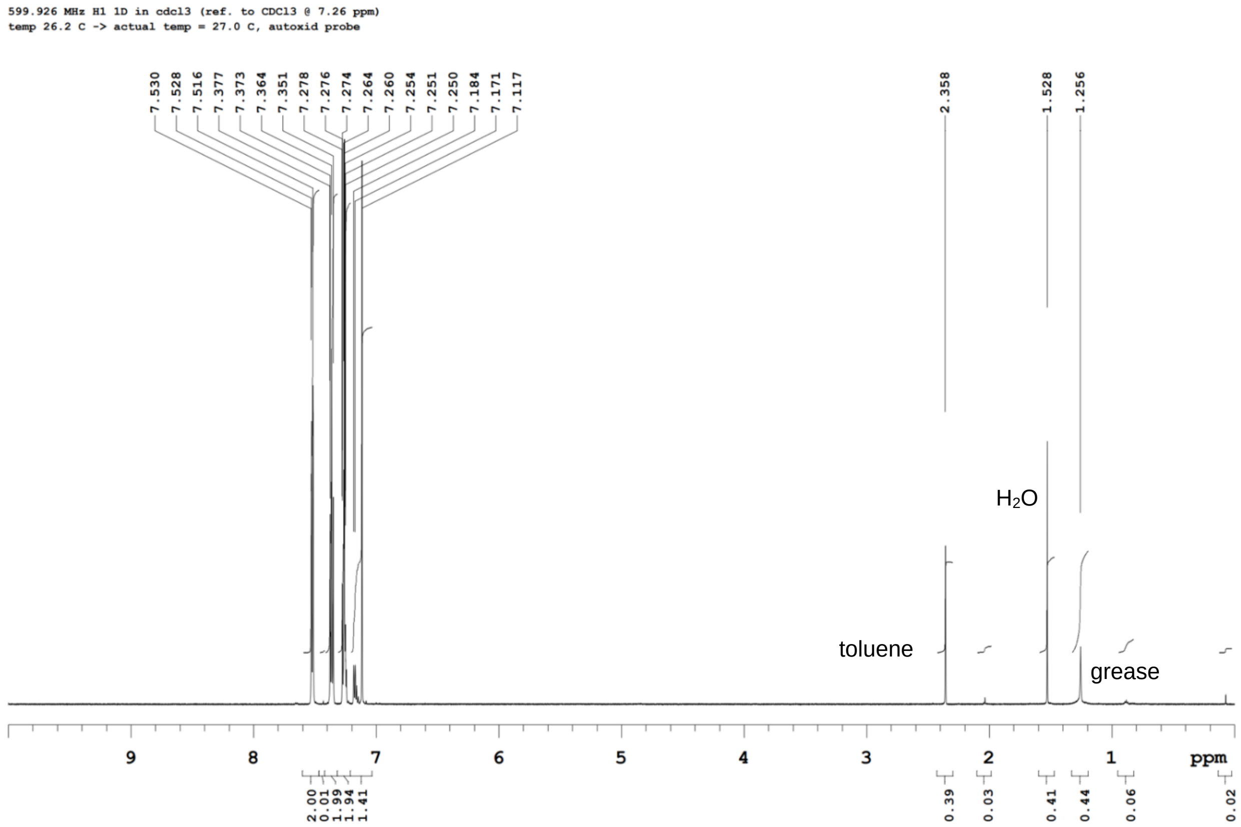

Figure S18. ^{1}H NMR of stilbene isomerization by ITO-NP for 16 h. The δ region from 10 to 0 ppm.

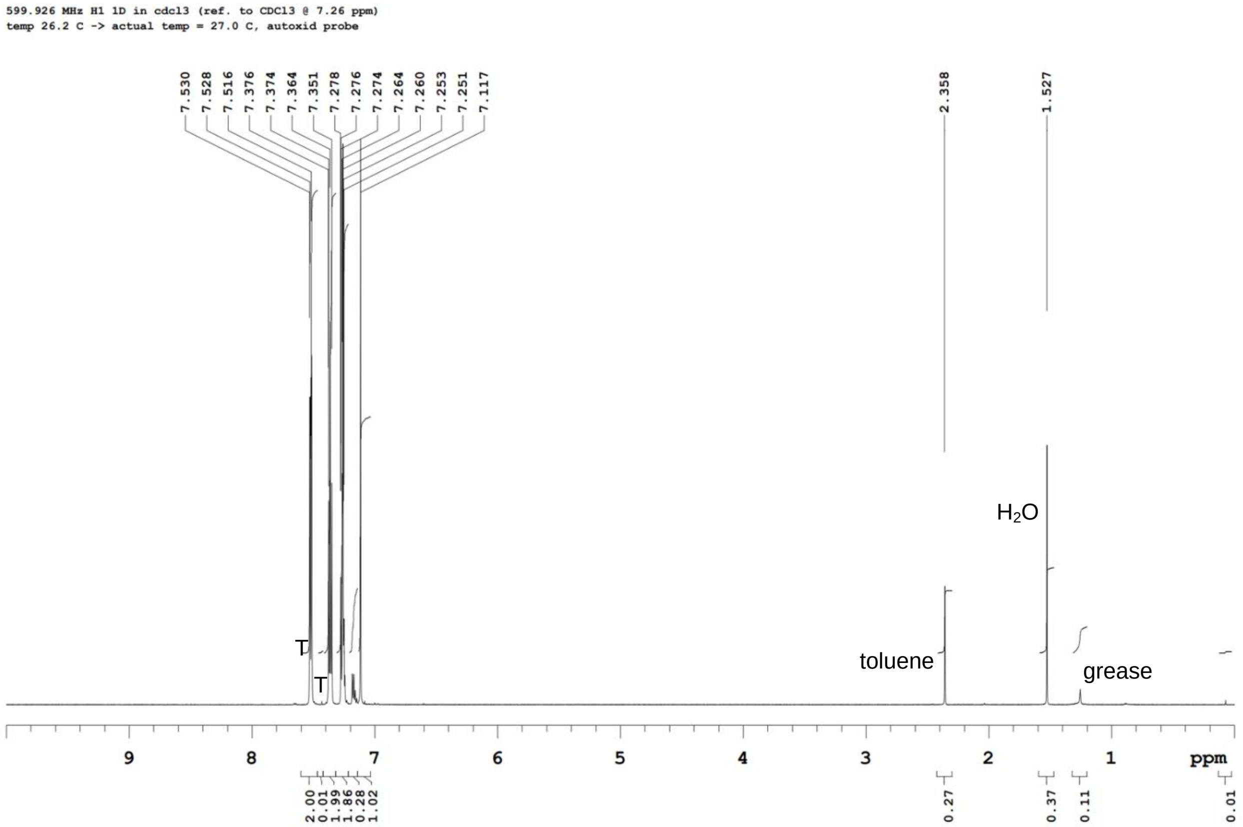

Figure S19. ^{1}H NMR of stilbene isomerization by ITO-NP for 48 h. The δ region from 10 to 0 ppm.

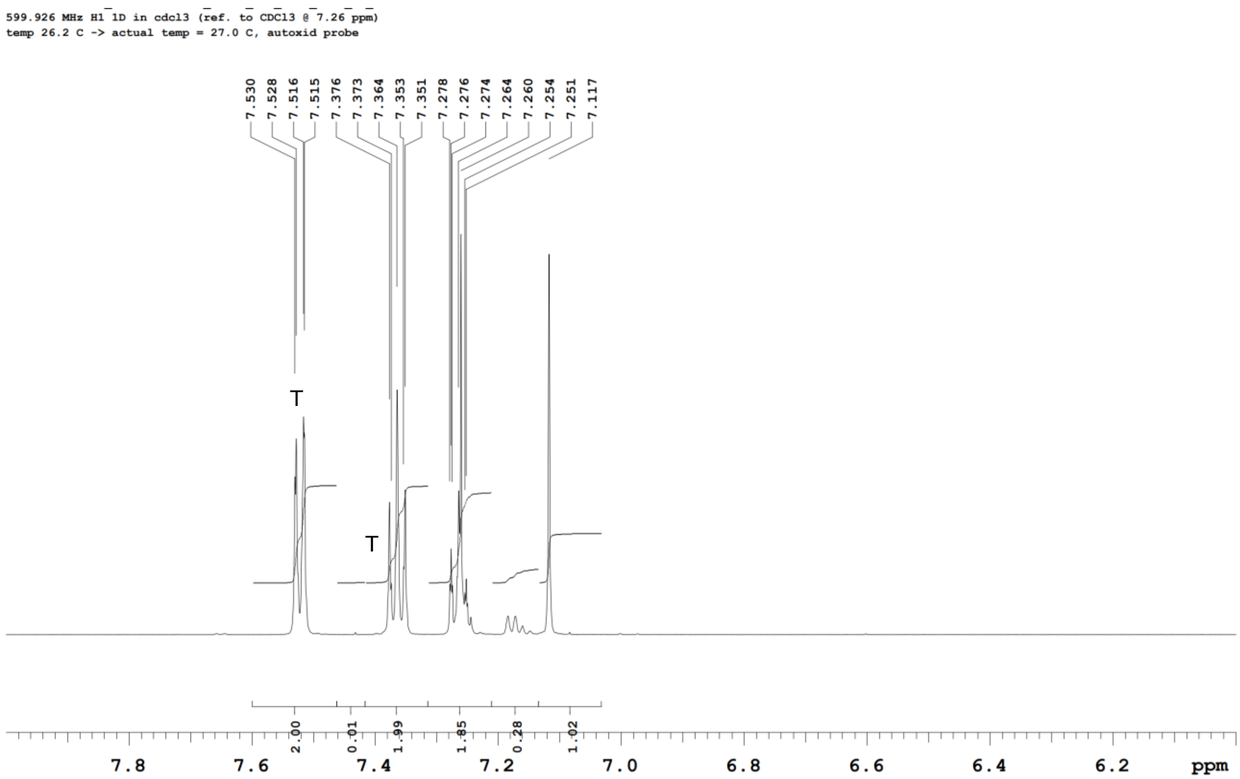

Figure S20. Magnified ^{1}H NMR of stilbene isomerization by new ITO-NP for 48 h. The δ region from 6 to 8 ppm showing ^{1}H NMR.

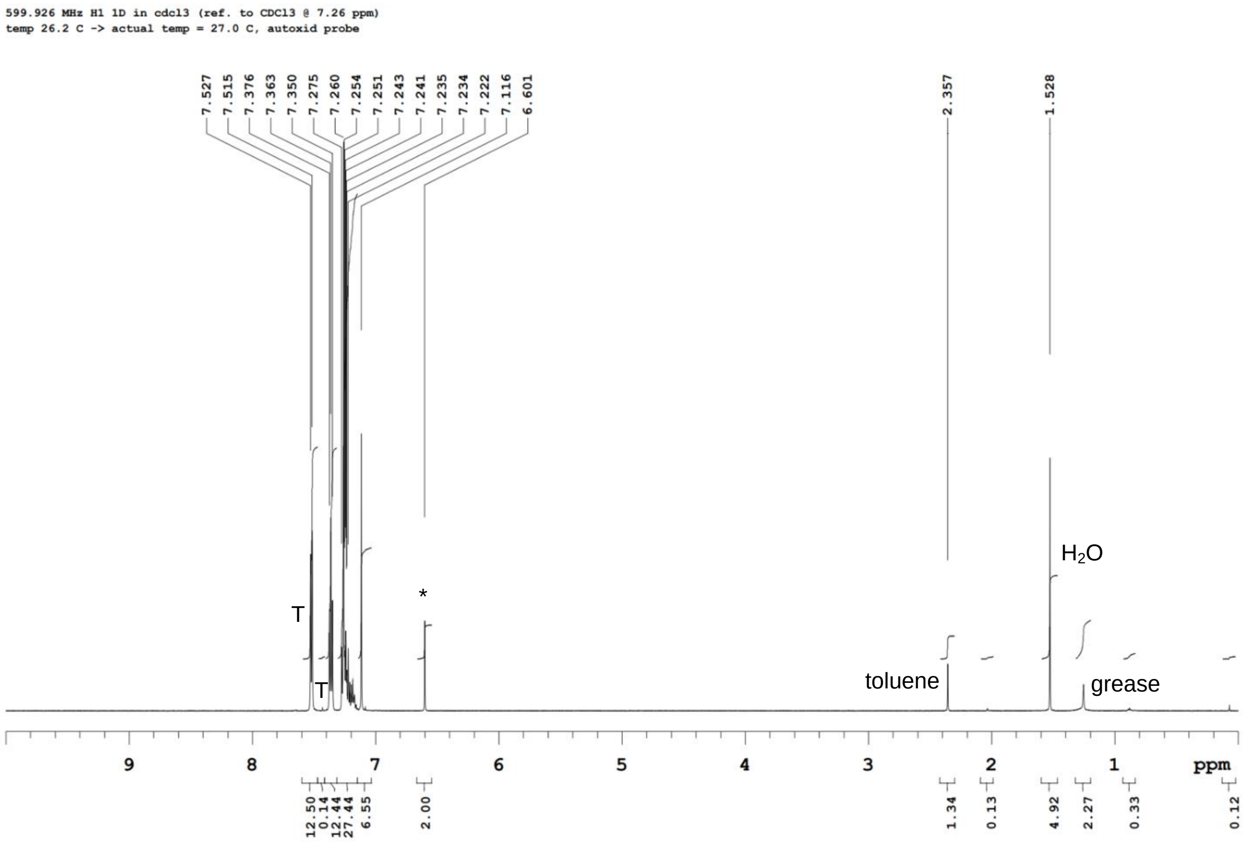

Figure S21. ^{1}H NMR of stilbene isomerization by 5-**1**-ITO-NP for 16 h. The δ region from 10 to 0 ppm showing ^{1}H NMR.

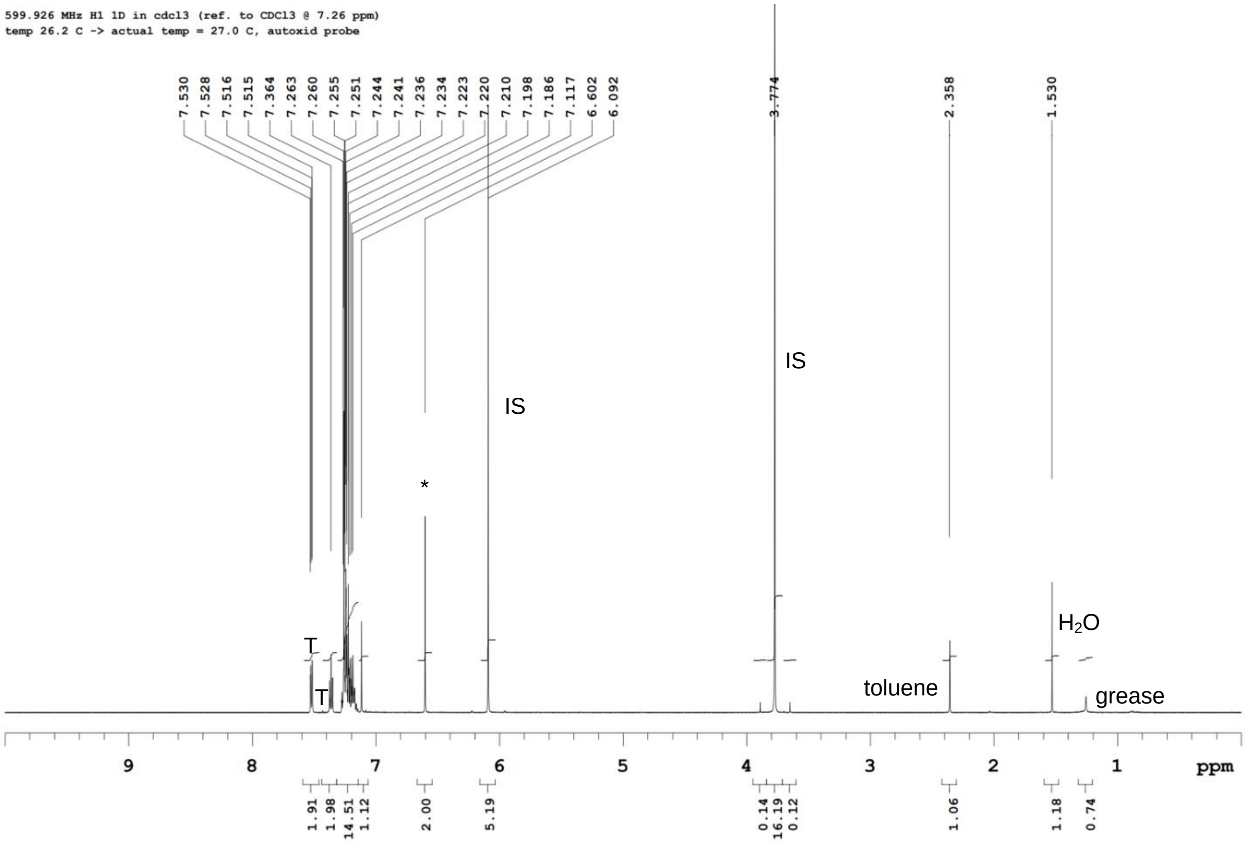

Figure S22. ^{1}H NMR of stilbene isomerization by 5-**1**-ITO-NP for 48 h. The δ region from 10 to 0 ppm showing ^{1}H NMR.

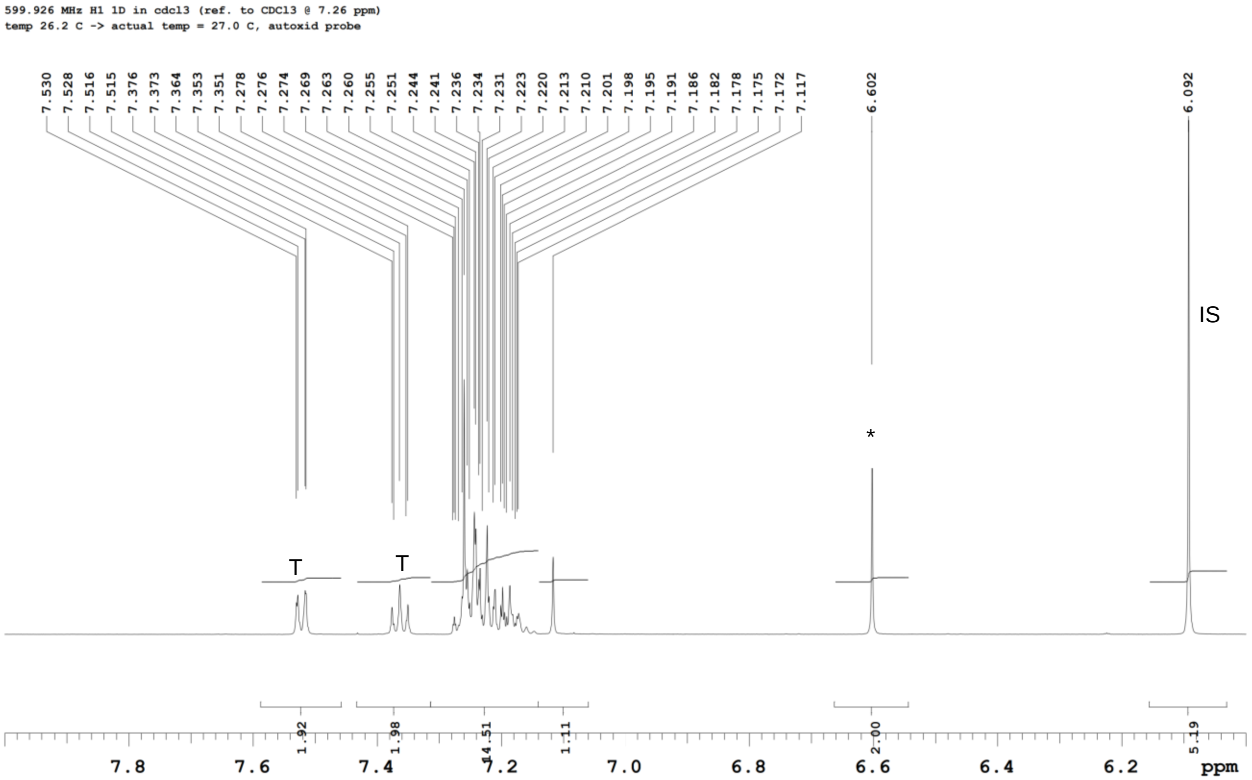

Figure S23. Magnified ^{1}H NMR of stilbene isomerization by 5-**1**-ITO-NP for 48 h. The δ region from 6 to 8 ppm showing ^{1}H NMR.

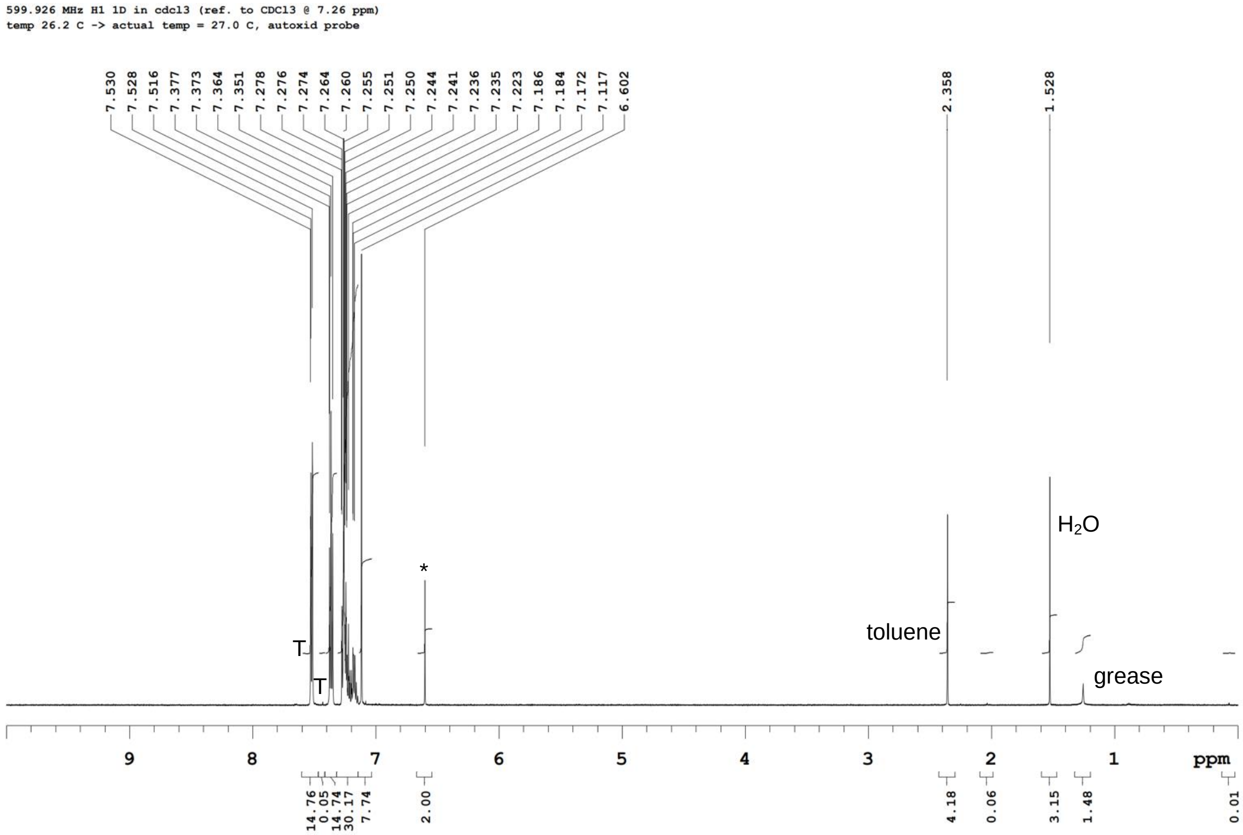

Figure S24. ^{1}H NMR of stilbene isomerization by 10-**1**-ITO-NP for 16 h. The δ region from 10 to 0 ppm showing ^{1}H NMR.

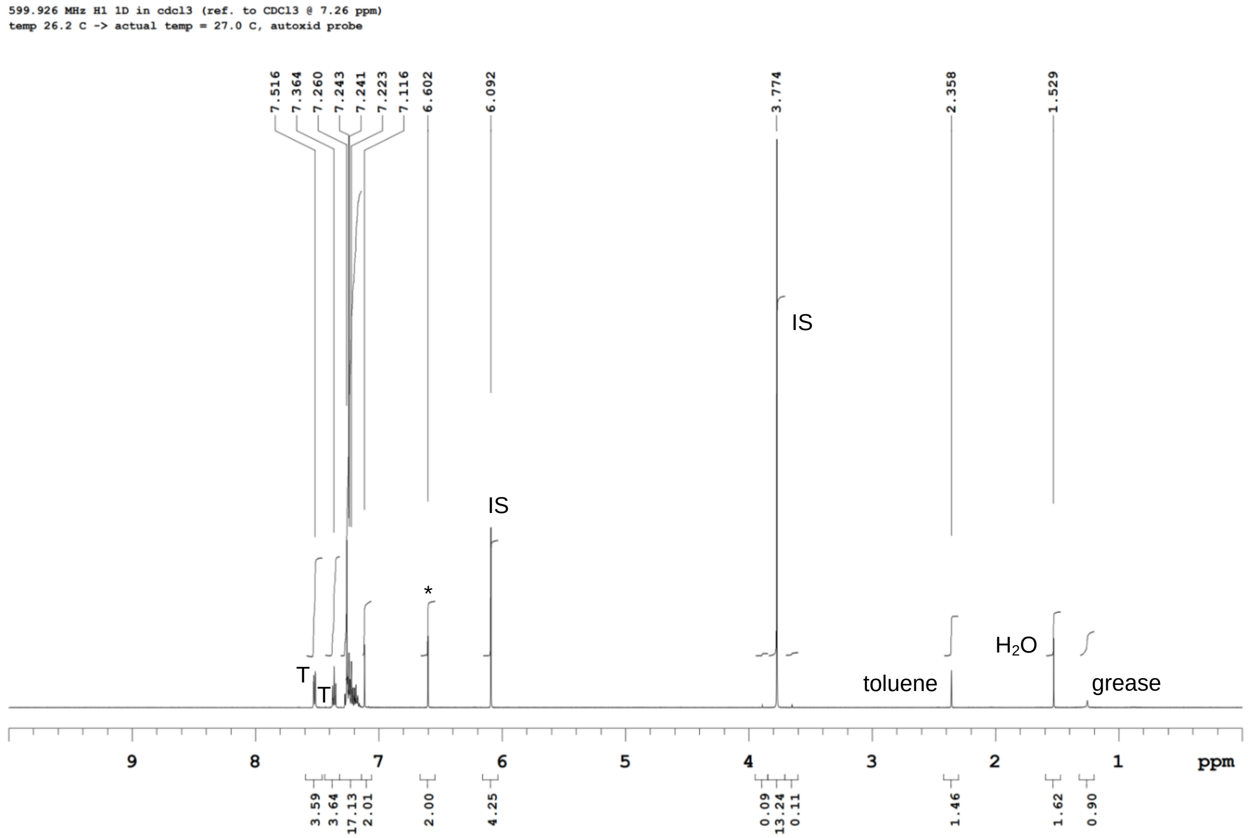

Figure S25. ^{1}H NMR of stilbene isomerization by 10-**1**-ITO-NP for 48 h. The δ region from 10 to 0 ppm showing ^{1}H NMR.

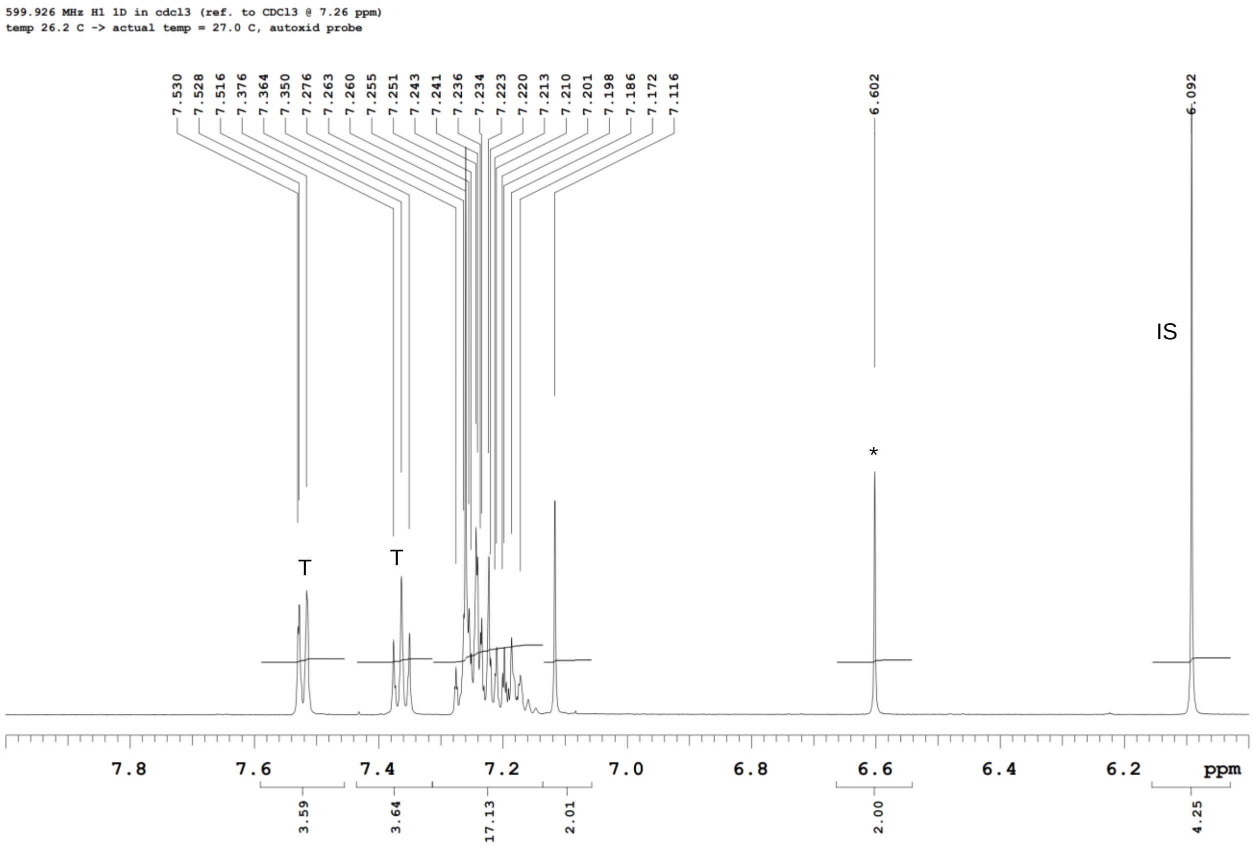

Figure S26. Magnified ^{1}H NMR of stilbene isomerization by 10-**1**-ITO-NP for 48 h. The δ region from 6 to 8 ppm showing ^{1}H NMR.

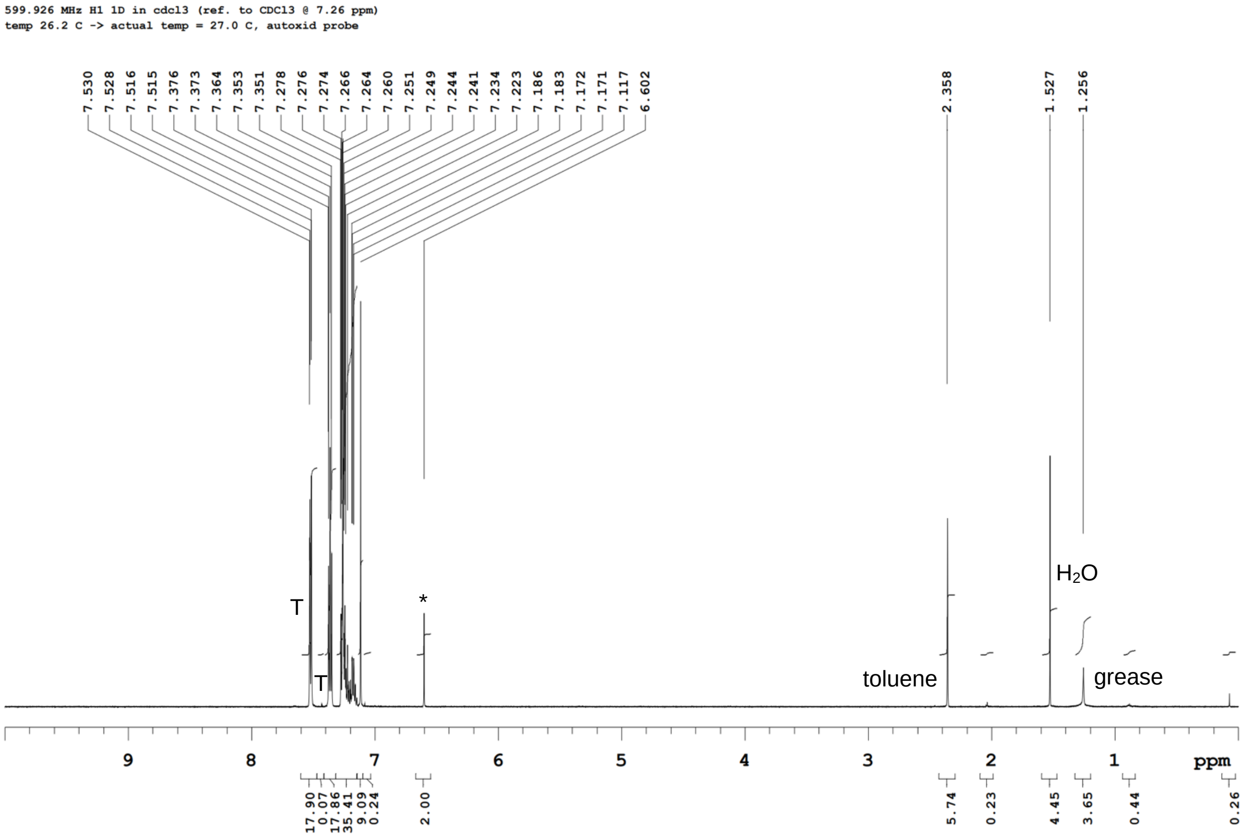

Figure S27. ^{1}H NMR of stilbene isomerization by 45-**1**-ITO-NP for 16 h. The δ region from 10 to 0 ppm showing ^{1}H NMR.

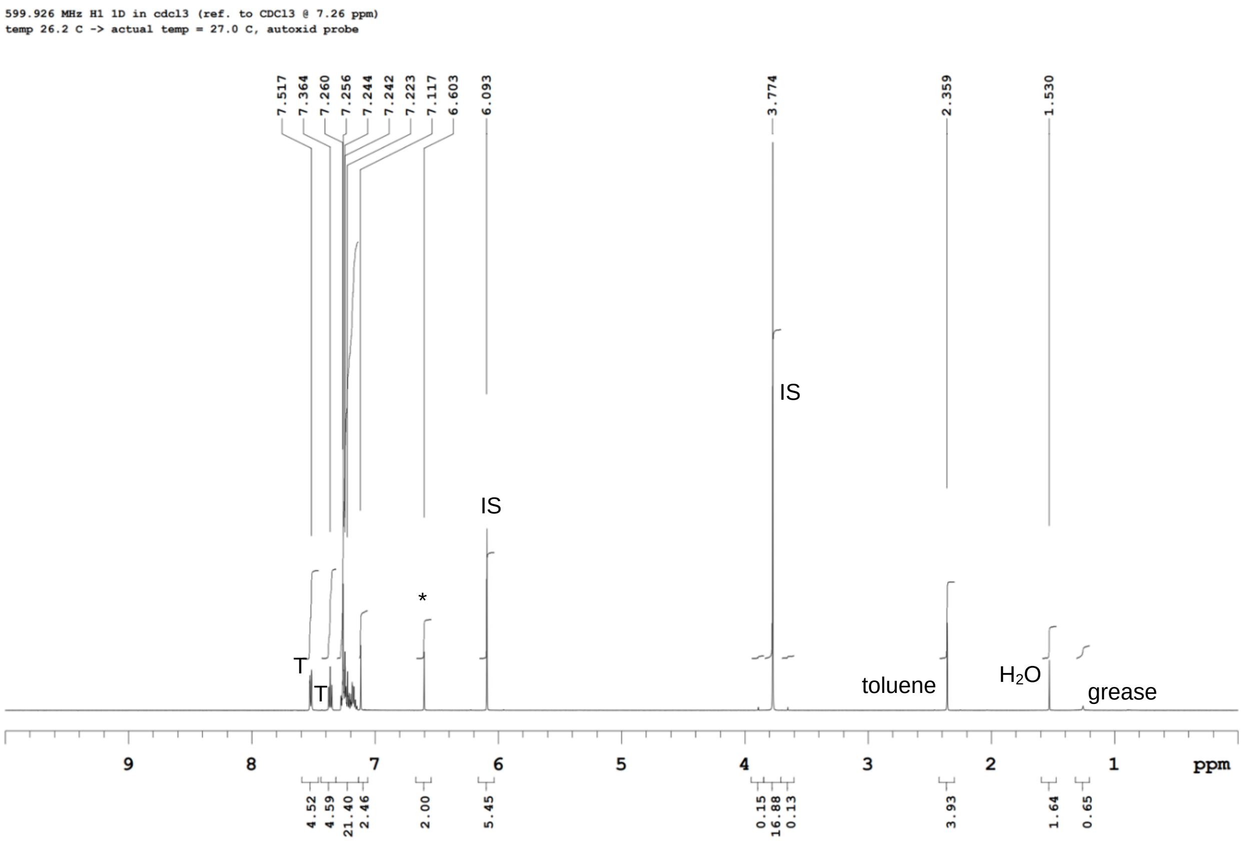

Figure S28. ^{1}H NMR of stilbene isomerization by 45-**1**-ITO-NP for 48 h. The δ region from 10 to 0 ppm showing ^{1}H NMR.

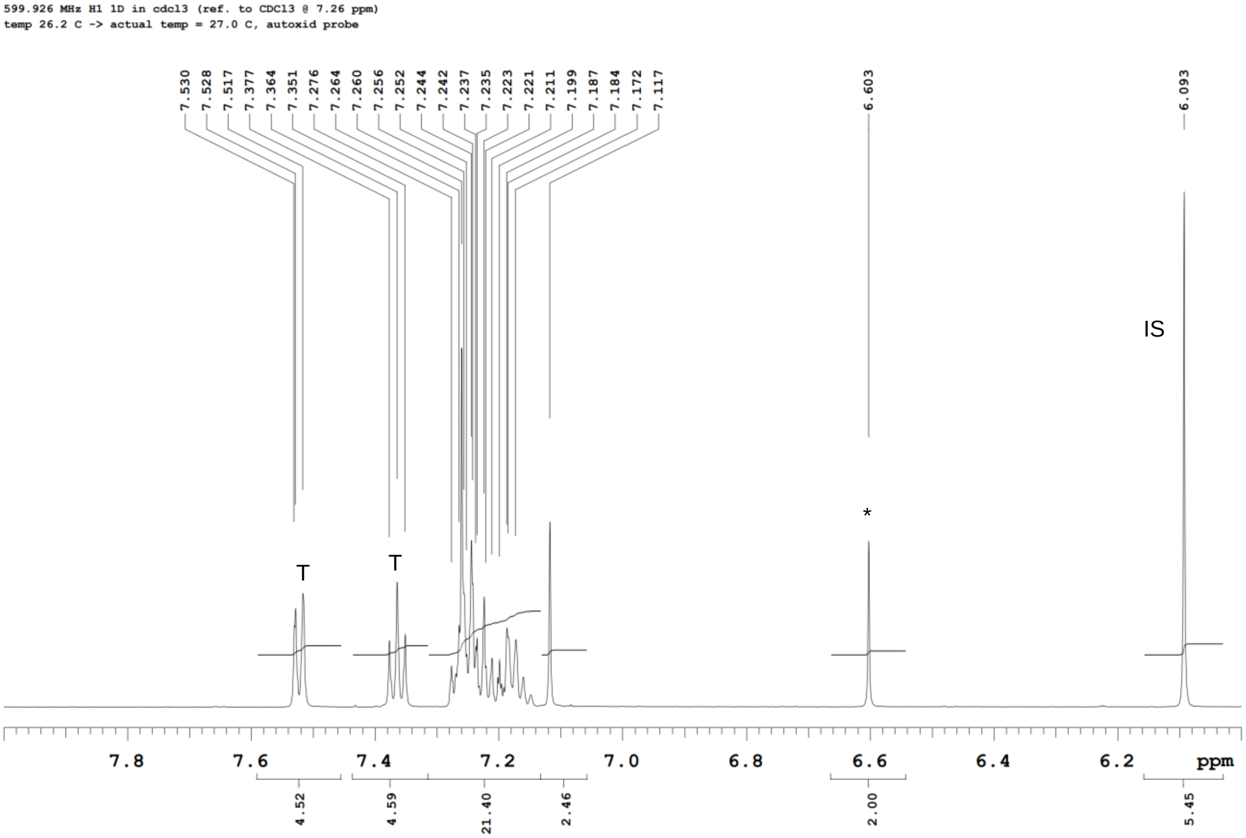

Figure S29. Magnified ^{1}H NMR of stilbene isomerization by 45-**1**-ITO-NP for 48 h. The δ region from 6 to 8 ppm showing ^{1}H NMR.

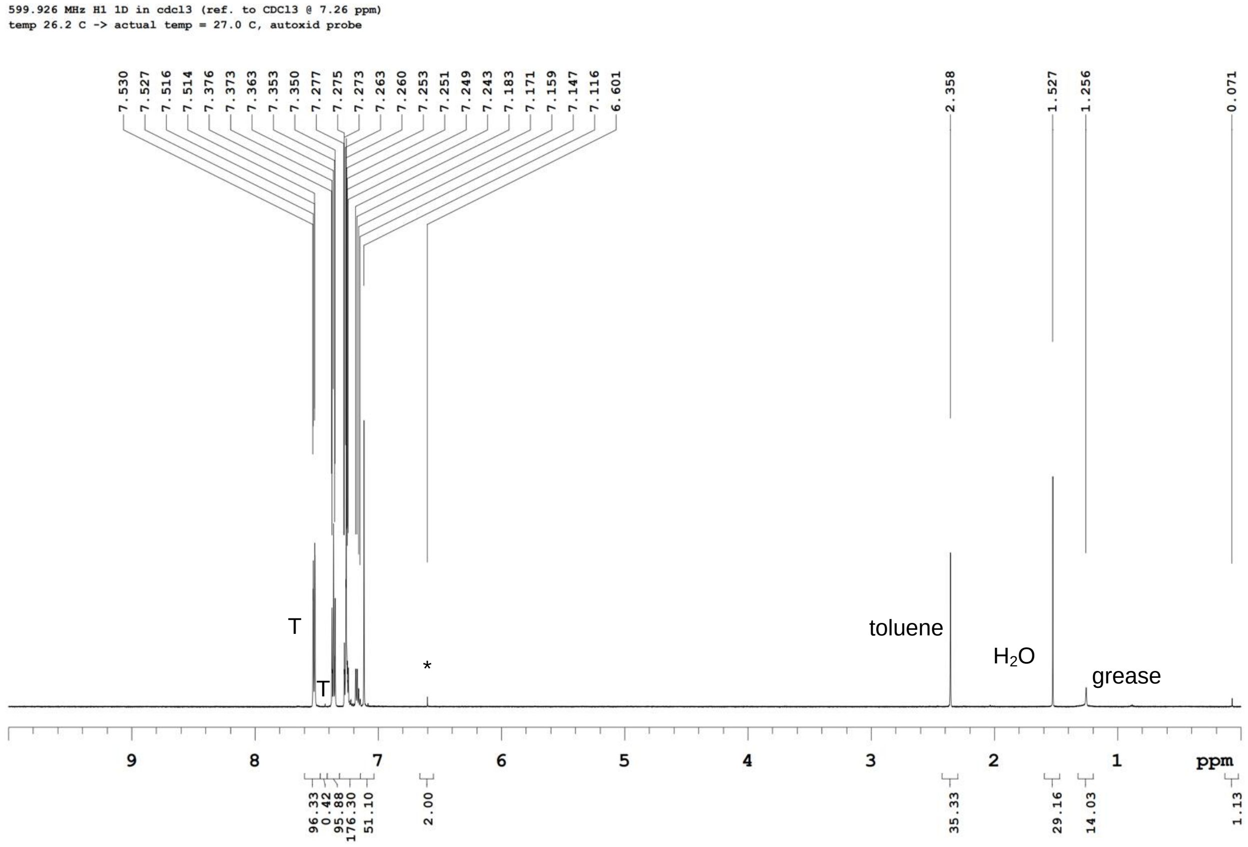

Figure S30. ^{1}H NMR of stilbene isomerization by the second run of 5-**1**-ITO-NP for 16 h. The δ region from 10 to 0 ppm showing ^{1}H NMR.

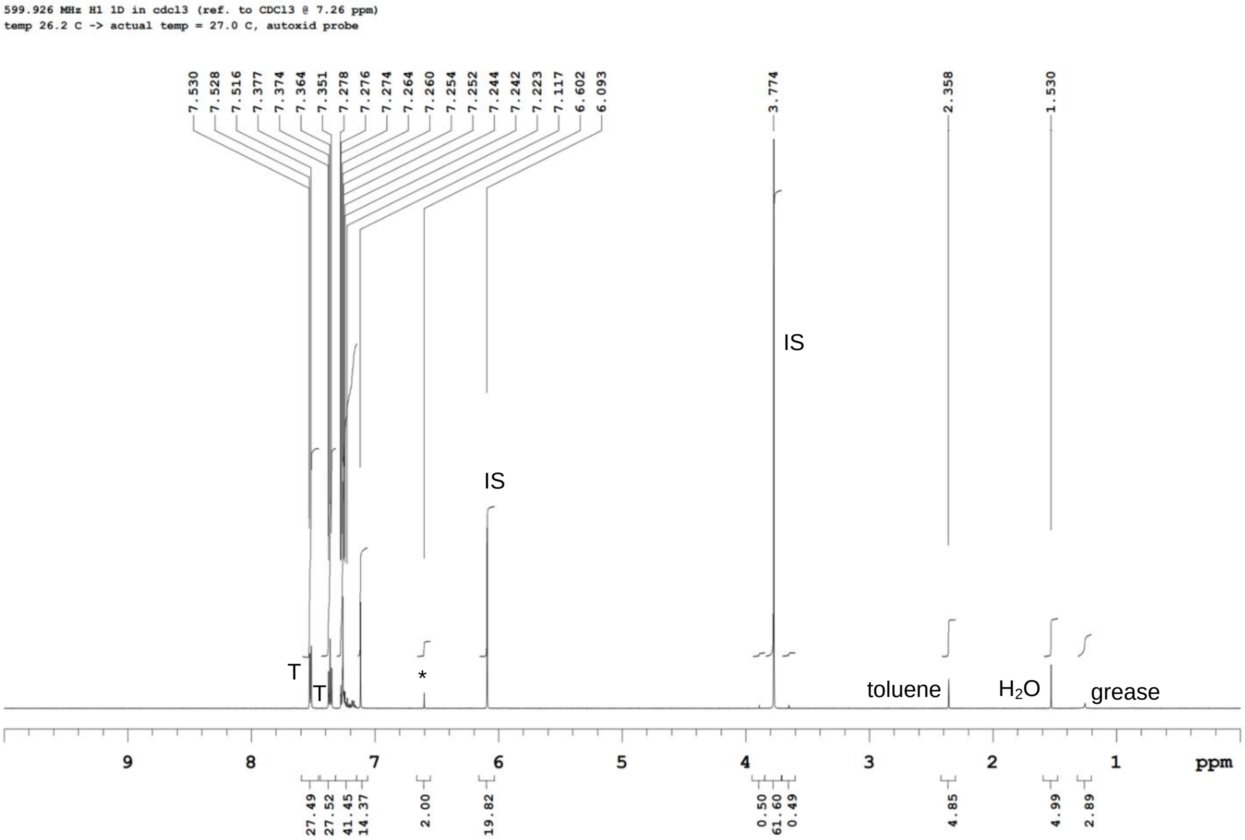

Figure S31. ^{1}H NMR of stilbene isomerization by the second run of 5-**1**-ITO-NP for 48 h. The δ region from 10 to 0 ppm showing ^{1}H NMR.

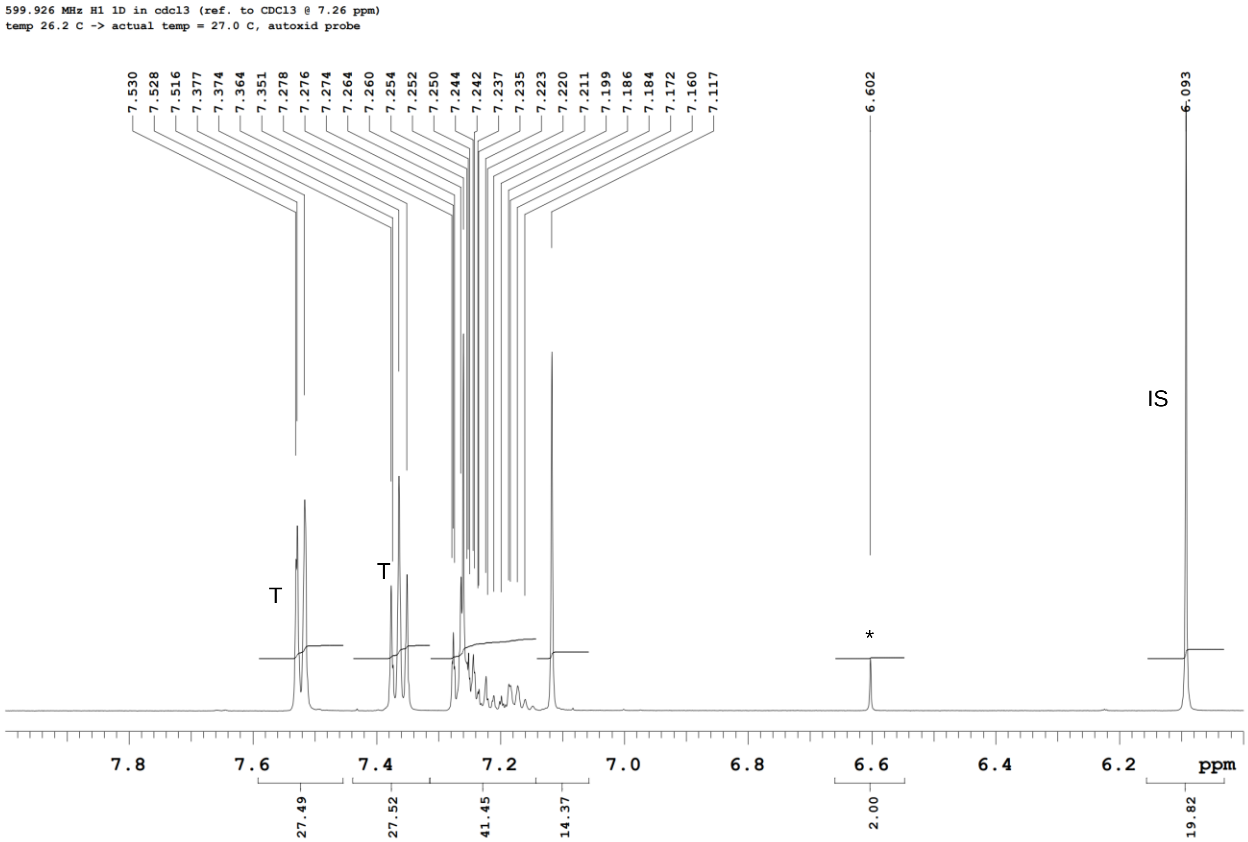

Figure S32. Magnified ^{1}H NMR of stilbene isomerization by the second run of 5-**1**-ITO-NP for 48 h. The δ region from 6 to 8 ppm showing ^{1}H NMR.

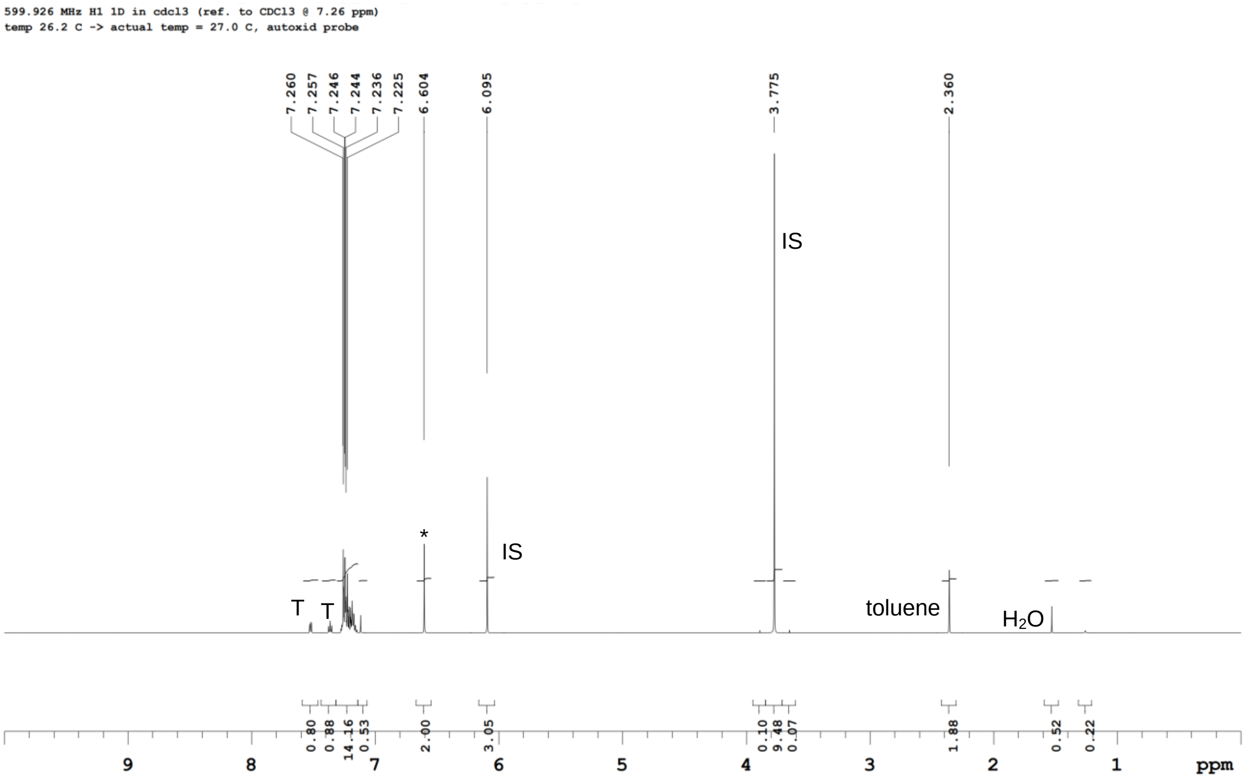

Figure S33. ^{1}H NMR of stilbene isomerization by new 5-**1**-ITO-NP for 88 h. The δ region from 10 to 0 ppm showing ^{1}H NMR.

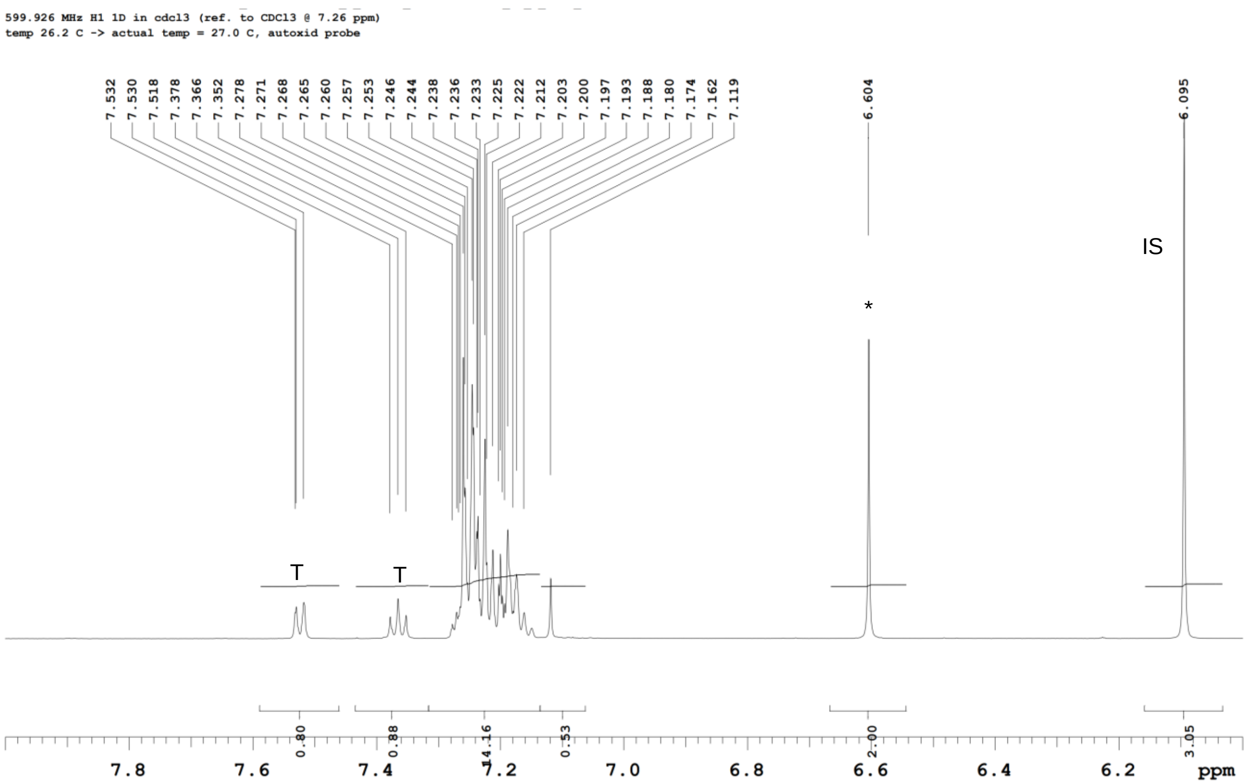

Figure S34. Magnified ^{1}H NMR of stilbene isomerization by 5-**1**-ITO-NP for 88 h. The δ region from 6 to 8 ppm showing ^{1}H NMR.

Crystallographic Experimental Details for 3CzImIPN (1)

Crystals suitable for solid-state structure determination were prepared by liquid–liquid diffusion of Et_2O into a DCM solution of **1**.

Table 1. Crystallographic experimental details.

A. Crystal Data	
formula	$C_{47}H_{27}N_7$
formula weight	689.75
crystal colour and habit[a]	yellow plate
crystal dimensions (mm)	0.16 × 0.10 × 0.03
crystal system	orthorhombic
space group	*Pbcn* (No. 60)
unit cell parameters[b]	
a (Å)	42.8659 (8)
b (Å)	11.9178 (2)
c (Å)	14.3275 (2)
V (Å^3)	7319.5 (2)
Z	8
ρ_{calcd} (g cm^{-3})	1.252
μ (mm^{-1})	0.596
B. Data Collection and Refinement Conditions	
diffractometer	Bruker D8/APEX II CCD[c]
radiation (λ [Å])	Cu Kα (1.54178) (microfocus source)
temperature (°C)	–100
scan type	ω and ϕ scans (1.0°) (5–10–15 s exposures)[d]
data collection 2θ limit (deg)	147.59
total data collected	112845 ($-52 \le h \le 53$, $-14 \le k \le 14$, $-17 \le l \le 17$)
independent reflections	7372 (R_{int} = 0.0651)
number of observed reflections (*NO*)	5922 [$F_o^2 \ge 2\sigma(F_o^2)$]
structure-solution method	intrinsic phasing (*SHELXT-2014*[e])
refinement method	full-matrix least-squares on F^2 (*SHELXL–2018*[f])
absorption-correction method	Gaussian integration (face-indexed)
range of transmission factors	1.0000–0.9079
data/restraints/parameters	7372/0/487
goodness-of-fit (*S*)[g] [all data]	1.031
final *R* indices[h]	
R_1 [$F_o^2 \ge 2\sigma(F_o^2)$]	0.0427
wR_2 [all data]	0.1074
largest difference peak and hole	0.152 and –0.210 e Å^{-3}

[a]Obtained by recrystallization from a dichloromethane/diethylether solution.

[b]Obtained from least-squares refinement of 9903 reflections with 7.70° < 2θ < 144.70°.

[c]Programs for diffractometer operation, data collection, data reduction, and absorption correction were supplied by Bruker.

[d]Data were collected with the detector set at three different positions: low-angle (detector 2θ = –33º) data frames collected using a scan time of 5 s, medium-angle (detector 2θ = 75º) frames using a scan time of 10 s, and high-angle (detector 2θ = 117º) frames using a scan time of 15 s.

[e]Sheldrick, G. M. *Acta Crystallogr.* **2015**, *A71*, 3–8. (*SHELXT-2014*)

[f]Sheldrick, G. M. *Acta Crystallogr.* **2015**, *C71*, 3–8. (*SHELXL-2018/3*)

[g]$S = [\Sigma w(F_o^2 - F_c^2)^2/(n - p)]^{1/2}$ (n = number of data; p = number of parameters varied; $w = [\sigma^2(F_o^2) + (0.0469P)^2 + 2.3571P]^{-1}$, where $P = [\mathrm{Max}(F_o^2, 0) + 2F_c^2]/3$).

[h]$R_1 = \Sigma||F_o| - |F_c||/\Sigma|F_o|$; $wR_2 = [\Sigma w(F_o^2 - F_c^2)^2/\Sigma w(F_o^4)]^{1/2}$.

Supplementary References

36. Rasu, L.; Amiri, M.; Bergens, S.H. Carbazole–Cyanobenzene Dyes Electrografted to Carbon or Indium-Doped Tin Oxide Supports for Visible Light-Driven Photoanodes and Olefin Isomerizations. *ACS Appl. Mater. Interfaces* **2021**, *13*, 17745–17752, https://doi.org/10.1021/acsami.1c05064

75. Kaewprachu, P.; Jaisan, C.; Klunklin, W.; Phongthai, S.; Rawdkuen, S.; Tongdeesoontorn, W. Mechanical and Physicochemical Properties of Composite Biopolymer Films Based on Carboxymethyl Cellulose from Young Palmyra Palm Fruit Husk and Rice Flour. *Polymers* **2022**, *14*, 1872, https://doi.org/10.3390/polym14091872.

76. Robb, M.A.; Cheeseman, J.R., Scalmani, G.; Barone, V.; Petersson, G.A.; Nakatsuji, H.; Li, X.; Caricato, M.; Marenich, A.V.; Bloino, J.; Janesko, B.G.; et al. Gaussian 16, Gaussian, Inc., Wallingford CT, 2016.77. Devlin, F.J.; Finley, J.W.; Stephens, P.J.; Frisch, M.J. Ab Initio Calculation of Vibrational Absorption and Circular Dichroism Spectra Using Density Functional Force Fields: A Comparison of Local, Nonlocal, and Hybrid Density Functionals. *J. Phys. Chem.* **1995**, *99*, 16883–16902, https://doi.org/10.1021/j100046a014.

78. McLean, A.D.; Chandler, G.S. Contracted Gaussian basis sets for molecular calculations. I. Second row atoms, *Z*=11–18. *J. Chem. Phys.* **1980**, *72*, 5639–5648, https://doi.org/10.1063/1.438980.
